# Combined acquisition of diffusion and T_2_*-weighted measurements using simultaneous multi-contrast magnetic resonance imaging

**DOI:** 10.1007/s10334-021-00976-3

**Published:** 2021-12-02

**Authors:** Nora-Josefin Breutigam, Matthias Günther, Daniel Christopher Hoinkiss, Klaus Eickel, Robert Frost, Mareike Alicja Buck, David A. Porter

**Affiliations:** 1 Imaging Physics, Fraunhofer Institute for Digital Medicine MEVIS, Max-von-Laue-Str. 2, 28359 Bremen, Germany; 2 Mediri GmbH, Heidelberg, DE Germany; 3 University of Bremen, Bremen, DE Germany; 4 Athinoula A. Martinos Center for Biomedical Imaging, Massachusetts General Hospital, Charlestown, MA USA; 5 Department of Radiology, Harvard Medical School, Boston, MA USA; 6 Imaging Centre of Excellence (ICE), University of Glasgow, Glasgow, Scotland

**Keywords:** Echo-planar imaging, Diffusion, Stroke

## Abstract

**Object:**

In this work, we present a technique called simultaneous multi-contrast imaging (SMC) to acquire multiple contrasts within a single measurement. Simultaneous multi-slice imaging (SMS) shortens scan time by allowing the repetition time (TR) to be reduced for a given number of slices. SMC imaging preserves TR, while combining different scan types into a single acquisition. This technique offers new opportunities in clinical protocols where examination time is a critical factor and multiple image contrasts must be acquired.

**Materials and methods:**

High-resolution, navigator-corrected, diffusion-weighted imaging was performed simultaneously with T_2_*-weighted acquisition at 3 T in a phantom and in five healthy subjects using an adapted readout-segmented EPI sequence (rs-EPI).

**Results:**

The results demonstrated that simultaneous acquisition of two contrasts (here diffusion-weighted imaging and T_2_*-weighting) with SMC imaging is feasible with robust separation of contrasts and minimal effect on image quality.

**Discussion:**

The simultaneous acquisition of multiple contrasts reduces the overall examination time and there is an inherent registration between contrasts. By using the results of this study to control saturation effects in SMC, the method enables rapid acquisition of distortion-matched and well-registered diffusion-weighted and T_2_*-weighted imaging, which could support rapid diagnosis and treatment of acute stroke.

**Supplementary Information:**

The online version contains supplementary material available at 10.1007/s10334-021-00976-3.

## Introduction

Multiple image contrasts in clinical MRI are usually acquired as a sequence of independent measurements. The scan time of each measurement can be reduced by various acceleration techniques.

A well-established acceleration technique is simultaneous multi-slice (SMS) imaging, in which multiple slices are excited and read out simultaneously. The single-slice signals can then be assigned to the individual slices in postprocessing. The basis for this method was proposed in the early days of modern MRI. In 1988, Souza and Müller independently published articles on the multi-slice technique, in which the simultaneously excited slices are additionally phase-labeled, also known as Hadamard labeling, to separate the acquired data during reconstruction [1, 2]. In the same year, Weaver proposed a multi-slice MRI method in which the simultaneously excited slices appear side by side due to an additional frequency shift [3]. Glover presented another encoding and reconstruction method in 1991. Similar to the previous techniques, this type of multi-slice MRI increased SNR but did not reduce acquisition time [4]. Then, in 2001, Larkman proposed a new reconstruction concept that effectively reduced scan time for the first time by using multiple receiver coils [5]. From then on, various improvements, e.g., by Breuer [6], Moeller [7], Feinberg [8], and Setsompop [9], paved the way for SMS in various scientific and clinical applications.

However, in many clinical 2D protocols the potential high acceleration cannot be fully exploited because the repetition time (TR) cannot be shortened further without reducing signal-to-noise ratio (SNR) or changing image contrast by an unacceptable amount. Low acceleration factors with multi-band RF pulses exciting two or three slices simultaneously may not be a problem, but at higher accelerations TR might become too short. This is particularly true for protocols with few slices. In the present work, we introduce a technique called simultaneous multi-contrast (SMC) imaging that uses SMS methodology to shorten overall examination time—while maintaining TR—by combining different scan types into a single acquisition. This technique offers new possibilities in clinical protocols where the examination time is a critical factor, the initial TR is too short for (high) SMS acceleration, and multiple image contrasts have to be acquired for diagnosis. SMC imaging can be used alone, but it is in combination with acceleration techniques such as in-plane acceleration and low-SMS acceleration that it will most likely reach its full potential.

In this study, SMC was used as a proof-of-concept to acquire diffusion-weighted (DW) and T_2_*-weighted (T_2_*W) images in a single scan. These two types of image contrast are often required in clinical examinations, most notably in the case of acute stroke, where diffusion-weighted imaging (DWI) is sensitive to acute infarct and T_2_*W imaging can be used to exclude intracranial hemorrhage and to detect microbleeds [10, 11]. In these studies, T_2_*W images are acquired using gradient-recalled echo (GRE) sequences, such as FLASH [12, 13] or single-shot, echo-planar imaging (ss-EPI) [14, 15]. There are several publications showing that GRE-EPI for T_2_*W imaging is an appropriate choice for the detection of microbleeds in acute cerebral stroke [16–20], even at a relatively course base resolution [20, 21]. Depending on the sequence type and protocol parameters, the scan times for these T_2_*W acquisitions range from tens of seconds to several minutes, with a corresponding variation in image quality and diagnostic value.

The sequence type used for SMC imaging in this study is based on readout-segmented, echo-planar imaging (rs-EPI), which is widely used for DWI [22], but its application to T_2_*W imaging is limited to preliminary work [23, 24]. Studies have demonstrated that rs-EPI for DWI can produce high-resolution DW images of the brain with a robust correction for motion-induced phase errors [25–27]. It has also been shown that the readout-segmented approach to DWI provides good visualization of acute brain ischemia [28]. In previous work, SMS has been used with rs-EPI in the conventional way to reduce the TR and consequently the scan time [29]. In contrast, this study introduces an application of the SMS method that maintains the rs-EPI TR and scan time, while simultaneously acquiring an additional T_2_*W image contrast.

The SMC imaging method presented here must consider the mutual influence of the different contrast excitations. In this study, we considered it particularly important to minimize the influence of the T_2_*W excitation on DW images, because of the low intrinsic signal-to-noise ratio (SNR) in the DW images [30]. For the T_2_*W images, the overall contrast difference between veins and grey and white matter (GM, WM) is important to detect hemorrhage. Although DW excitation has an effect on the T_2_*W images and leads to visible contrast changes in the main acquisition scheme used in this work, we show that the contrast difference between veins and GM and WM is largely preserved. The chosen parameters also result in a loss of SNR in the T_2_*W images compared to the equivalent stand-alone acquisition, but this is mitigated by the option to acquire multiple signal averages for the T_2_*W acquisition. Therefore, for this study, we accepted higher impact and contrast changes in the T_2_*W data and prioritized the objective of preserving the SNR and contrast in the DW images.

The proposed method provides an inherent anatomical registration between the contrasts by avoiding the effect of subject motion between multiple scans. Furthermore, the method performs a simultaneous readout of data for the two contrasts, which ensures that the images exhibit the same distortions, resulting in a further improvement in spatial alignment. This close anatomical match between the multi-contrast images could improve radiological interpretation and provide particular benefits when quantitative methods are used that rely on an accurate voxel-to-voxel registration between contrasts. In particular, for the contrast combination of DW and T_2_*W used in this study, there are proposals to combine quantitative information from diffusion tensor imaging (DTI) and quantitative susceptibility mapping (QSM) [31]. Similarly, there are studies which use information from both DW fMRI and BOLD fMRI [32].

## Materials and methods

### Overview of SMC method

The general concept of SMC imaging is to excite multiple slices and induce slice-specific image contrast before the slice signals are read out simultaneously. The slice-specific image contrast can be generated by varying the flip angles and magnetization-preparation modules that operate at each slice position. Previous work has investigated the simultaneous acquisition of orthogonal slices with different TEs for the purpose of localizer scans [33]. The SMC method in the current study concerns the simultaneous acquisition of multi-contrast data from multiple slice positions for diagnostic purposes.

Similar to the well-known SMS technique, auto-calibration signals (ACS) are required to separate the single-contrast signals from collapsed multi-contrast data using the slice-GRAPPA algorithm [9]. In principle, the method can be used to combine a range of contrast types, but in practice the choice will be influenced by the level of signal-change effects. Combinations in which one contrast requires a lower excitation flip angle can be a favorable choice. The initial implementation of SMC used in this study focuses on the combined acquisition of DWI and T_2_*W imaging. A preliminary version of this work has been presented in abstract form [34–37].

### SMC sequence design

In general, the sequence design for SMC imaging must take the mutual influence of the contrast excitations into account. This is influenced by the time between excitations (slice iteration scheme), the chosen TR and the chosen flip angles. These magnetization effects can be predicted with a Bloch simulation. For the selected contrast combination of DW and T_2_*W, it is sufficient to consider T_1_ effects. The slice iteration scheme should be chosen to achieve a balanced compromise between mutual signal-change effects and scan time. For the chosen contrast combination in this study, it was particularly important to preserve SNR in gray matter (GM) and white matter (WM) for the DW contrast. Therefore, the influence of the T_2_*W excitation signal from the DW slice should be as small as possible.

DW rs-EPI sequence with blipped CAIPIRINHA [9, 38] was modified to acquire signals from two slice positions simultaneously. The slices were each subjected to a different magnetization preparation and were excited at separate times with different excitation pulses to obtain varying image contrasts. The first slice was used to provide the DW contrast, while the second slice provided the T_2_*W contrast.

**Fig. 1:**
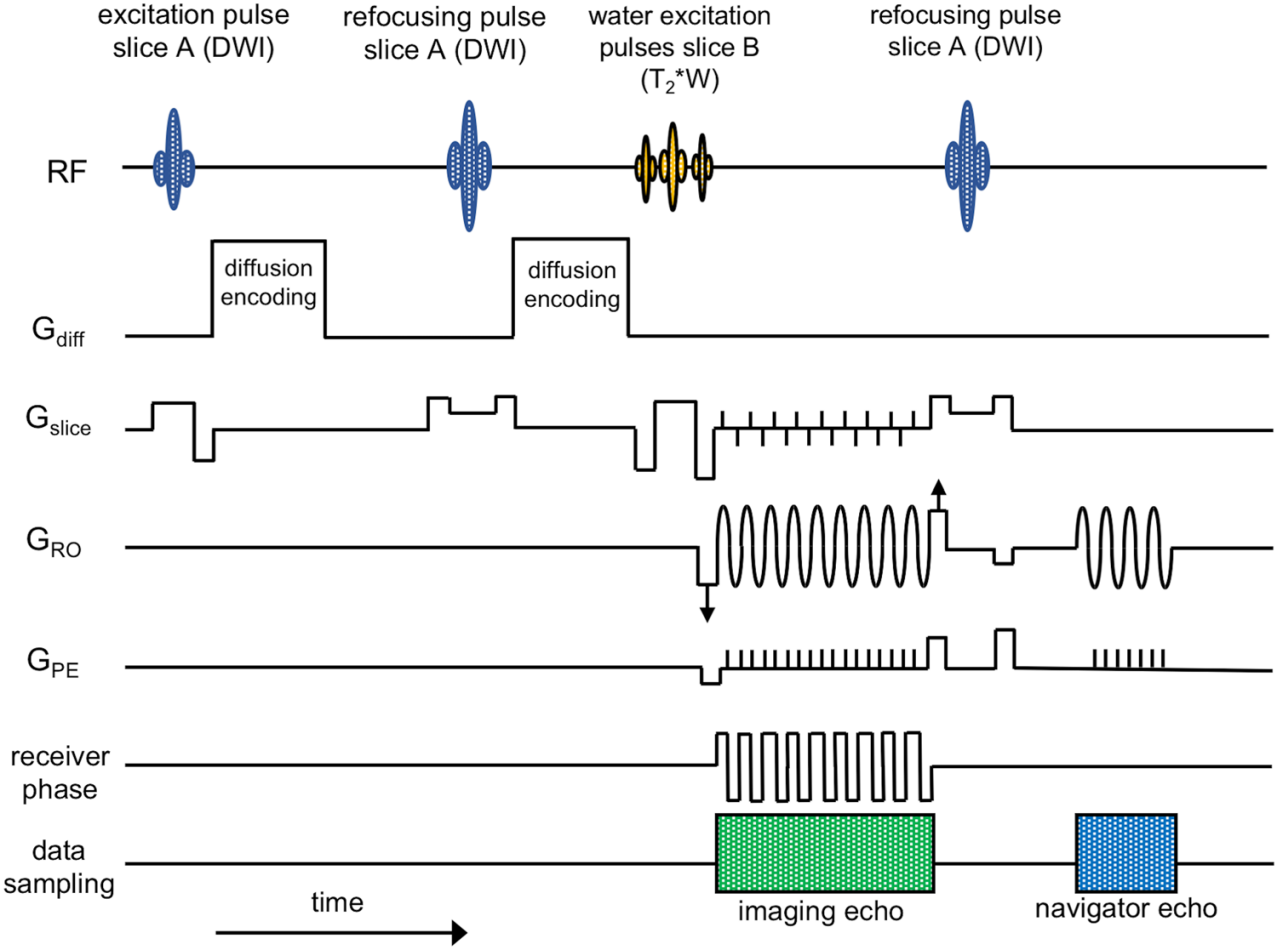
Pulse sequence diagram of the rs-EPI sequence for SMC imaging. Data are acquired at two slice positions at the same time with one slice (**A**) generating DW-while the other slice (**B**) provides T_2_*W image contrast. Firstly, slice A is excited and DW preparation is applied. Then slice B is excited before both signals are read out simultaneously using rs-EPI with a variable amplitude encoding gradient in the readout (*G*_RO_) direction (labelled with arrows) and a blipped phase-encoding gradient (*G*_PE_). A blipped-CAIPIRINHA gradient scheme along the slice-select (*G*_slice_) direction is used in conjunction with receiver phase modulation to shift the T_2_*W image by half a FOV in the phase-encoding direction. Finally, an RF refocusing pulse is applied to slice A only to generate a 2D navigator signal for phase correction. Note that a binomial water-excitation pulse was used in this study for the T_2_*W excitation

The pulse-sequence diagram is shown in Fig. 1. Firstly, slice A is excited and DW preparation is applied. Then slice B is excited before both signals are sampled simultaneously using rs-EPI with a sinusoidal readout gradient [22], a variable-amplitude encoding gradient in the readout (RO) direction (labelled with arrows in Fig. 1), and a blipped phase-encoding gradient (*G*_PE_). The variable-amplitude encoding gradient is applied to define an offset along the RO direction which varies from shot-to-shot to sample a different readout segment, each covering the complete range of *k*_*y*_ values and a restricted range of *k*_*x*_ values. In the current SMC implementation involving two slices, a blipped-CAIPIRINHA gradient scheme along the slice (*G*_slice_) direction is used in conjunction with receiver phase modulation to shift the T_2_*W image by half a field-of-view (FOV) in the phase-encoding (PE) direction relative to the DW image. Finally, a radiofrequency (RF) refocusing pulse is applied only to slice A to generate a low-resolution 2D navigator signal for phase correction of the DW image data during image reconstruction. Although not plotted in the sequence scheme in Fig. 1, spoiling gradients followed each readout to avoid the development of additional unwanted signal coherences.

The implementation of the sequence used in this work relies on gradient reversal [39] to suppress fat signals in the DW acquisition. To avoid fat signal in the T_2_*W images, a binomial water-excitation pulse was used. SMC acquisitions can be performed for parallel slices in all orientations; here, images were acquired in transverse and coronal orientations (Fig. S1).

To test which ACS data work best for separating the slice-specific signals, ACS data were acquired as two complete image volumes, the first corresponding to the DW component of the sequence with a nominal *b*-value of 0 s/mm^2^ (*b*_0_) and the second corresponding to the T_2_*W portion of the sequence. In both cases, only the central readout segment was acquired.

### Inline image reconstruction

Image reconstruction was performed using the slice-GRAPPA algorithm [9] with inter-slice leakage artefact reduction [40], also referred to as split slice-GRAPPA, which can be directly adapted for SMC. The algorithm was implemented in the vendor’s proprietary Image Calculation Environment (ICE) to allow execution at the scanner. The computation time was reduced by parallelization. A 2D kernel or weights-set was fitted to each slice of the ACS data. This was done with either the *b*_0_ ACS data alone, the T_2_*W ACS data alone, or with a combination of both. In the simplest form of SMC, where only two slices are acquired simultaneously, the slice-GRAPPA weights for the separation of the FOV-shifted T_2_*W data are calculated from ACS slices that are also shifted by FOV/2. The weight-sets calculated from the b_0_ ACS data were used for all *b*-values of the DW data. After calculating the weights-sets, the collapsed SMC data were convolved with the corresponding kernels to provide separated DW and T_2_*W data for each single coil.

An additional 2D non-linear navigator correction was applied to the DW data to correct for motion-induced phase errors [22, 27]. The CAIPIRINHA FOV shift of the T_2_*W slices was also reversed. Finally, the separated and reconstructed images from the individual coil channels were combined using the spatial matched filter (SMF) method [41, 42].

### Experiments

All experiments were performed on a 3 T whole-body MR scanner (MAGNETOM Skyra, Siemens Healthcare GmbH, Germany) using a 20-channel head coil. Dedicated pulse sequences and image reconstruction modules were developed in C+ +  using the manufacturer’s proprietary development tools.

Scans were performed on a spherical water-based phantom and with five healthy subjects (aged 26–42, one female). All subjects provided written informed consent prior to scanning and the study was run under a general protocol for pulse-sequence development approved by the local ethics committee.

All data were acquired using the dedicated rs-EPI sequence shown in Fig. 1. For comparison, single-contrast DW or T_2_*W images were acquired separately. The choice of acquisition parameters for the single-contrast data was entirely determined by the constraints of the SMC protocol, so that the timing of the single-contrast acquisitions matched those of the SMC acquisitions as closely as possible. This ensured, as much as possible, that the differences examined did not depend on other differences that might arise due to measurement parameters and sequence properties.

DW was applied using a three-scan, trace-weighted (TrW) acquisition with three orthogonal diffusion directions using nominal b-values of 0 s/mm^2^ and 1000 s/mm^2^. A (3 × 3) split slice-GRAPPA kernel size was used for reconstruction. The matrix size for the 2D navigator in the DW scan was 29 × 64 (RO × PE). Acquisition parameters (single-contrast and SMC) can be retrieved from Table 1. For these measurements, GRAPPA for in-plane acceleration was performed with an acceleration factor of two. The gradient-reversal technique to suppress fat signals results in prolonged RF pulse durations of 7.68 ms and 5.12 ms for the slice-selective excitation and refocusing pulses, respectively. Six pre-scans, each lasting one TR, were performed at the start of the measurement: two magnetization-preparation scans (one at the start of the measurement, and one immediately before the image-data acquisition to account for different RF pulse timing during the ACS acquisitions); a non-phase-encoded reference scan for Nyquist ghost phase correction; an ACS acquisition for in-plane GRAPPA data processing; and the acquisition of two ACS scans for slice-GRAPPA, corresponding to a DW b_0_ scan and a T_2_*W scan, respectively.

**Table 1:** Acquisition parameters ^**a,b**^

EPI sequence	Resolution (mm^3^)	FOV (mm^3^)	Matrix	Readout segments	ES (ms)	TR (ms)	TE_DW_ / TE_T2*W_ (ms)	# b = 0/ Diff. dir	Flip angle T_2_*W (°)	in-plane GRAPPA	Scan time (min)
rs-EPI DW single-contrast	1.0 $\times$ 1.0 $\times$ 3	256 $\times$ 256 $\times$ 150/256 $\times$ 256 $\times$ 120	256 $\times$ 256 $\times$ 20/256 $\times$ 256 $\times$ 10	9	0.34	4500/2250	81.5/–	1/3	–	2	3:09^**c**^/1:35^**c**^
rs-EPI T_2_*W single-contrast	1.0 $\times$ 1.0 $\times$ 3	256 $\times$ 256 $\times$ 150/256 $\times$ 256 $\times$ 120	256 × 256 × 20/256 × 256 × 10	9	0.34	4500/2250	–/24.8	–	20°	2	3:09^**c**^/1:35^**c**^
rs-EPI SMC	1.0 $\times$ 1.0 $\times$ 3	256 $\times$ 256 $\times$ 150/256 $\times$ 256 $\times$ 120	256 × 256 × 20/256 × 256 × 10	9	0.34	4500/2250	81.5/24.8	1/3	20°	2	3:09/1:35

^a^All protocols, if applicable, acquired with *b* = 1000 s/mm^2^

^b^Gaps between slices of 4.5 mm for 20 slices, and 9.0 mm for 10 slices were used

^c^Scan time matched to that of rs-EPI SMC to maintain signal comparison between sequences

Scan time without additional ACS data and preparation for SMC (same TR) = 2:56 min/1:28 min

*EPI* echo planar imaging, *ES* echo spacing

Compared to the standard DW rs-EPI sequence, the introduction of the additional water-excitation pulse for the T_2_*W images resulted in a DWI TE increase of 6 ms and a corresponding increase of 120 ms in the minimum TR for 20 slices.

Based on a preliminary analysis of mutual saturation effects between the RF pulses used for each contrast type, the distance between the two simultaneously acquired slices was chosen to be half of the slice FOV. For the chosen TR value of 4500 ms for in vivo measurements, the time between the excitation of the DW scan and the subsequent excitation for the T_2_*W scan at the same slice position was 2307 ms. The corresponding time between the excitation for T_2_*W imaging and the subsequent excitation for DWI was 2193 ms.

The additional excitation of each slice leads to signal saturation effects. To experimentally determine this signal changes, measurements with two slice positions were performed on the water-based phantom and in vivo with one subject. The in vivo measurements here were performed with two different slice iteration schemes. In slice iteration scheme A, the time between excitation of the DW scan and the subsequent T_2_*W scan was approximately ½ TR. In slice iteration scheme B, the time between excitation of the DW scan and the subsequent T_2_*W scan was approximately ¾ TR, and ¼ TR vice versa. One slice was positioned outside the phantom or head to prevent the effects of potential cross-talk between slices. Acquisitions were performed with both SMC and the corresponding single-contrast sequences. However, no in-plane acceleration was applied, resulting in a prolongation of TEs compared to the other acquisitions (TE_DW_/TE_T2*W_ = 128.6/46.5 ms). For the measurements in vivo, the TR was 4500 ms and the flip angles were 5°, 20°, and 84° (which corresponds to a T_2_*W Ernst angle at TR = 4500 ms and a *T*_1_ value of 2000 ms as a compromise between tissue and CSF *T*_1_ values). For the phantom measurements, the TR was 1500 ms and the *T*_2_*W flip angles were in the range of 5°–90° with an initial increment of 5°, and an increment of 10° from 20° upwards. The shorter TR time for phantom measurements was used because the *T*_1_ relaxation time of the phantom (approximately 290 ms) is shorter than the *T*_1_ relaxation times of the different brain tissues. *T*_1_ maps were generated using a gradient-echo, slice-selective, inversion-recovery sequence with TR = 10 s. B_1_ field maps were determined with a 2D EPI sequence using the double-angle method (DAM) (43) with $\alpha$ = 60° and 2 $\alpha$ = 120°.

SMC can theoretically be combined with conventional SMS acceleration. However, a shorter TR for SMC leads to higher signal changes. To explore the potential impact of this, data were acquired with half TR (10 slices) in four subjects to analyze the influence on mutual signal-change effects on image contrast compared to SNR loss due to shortened TR only.

Finally, since the T_2_*W contrast is affected by the influence of DW excitation, the vein-tissue contrast of the T_2_*W SMC images is compared with the results of standard clinical protocols using a 2D FLASH measurement with a duration of 2:16 min and a GRE-EPI sequence with a duration of 54 s.

### Numerical simulations

The mutual signal-change effects of the two-slice data were simulated for the same range of excitation flip-angles for the T_2_*W slice as for the phantom scans and for the measurements in vivo. To allow comparison with experimental data, *T*_1_ and *B*_1_ values determined from the phantom scans and images acquired in vivo were used for the Bloch simulations to estimate the mutual signal-change effects.

### Data processing and analysis

A comparison between simulated and measured signal-change effects was performed by calculating the mean and standard deviation of the percentage signal difference between SMC and the corresponding single-contrast data from: (a) simulations with *T*_1_ and *B*_1_ maps in the phantom and in vivo; and (b) experimental data obtained from two slices, with one slice outside the imaged object in each case. For the scans in vivo, tissue types WM and GM were analyzed separately because of the different *T*_1_ values. To analyze the influence of the slice-GRAPPA reconstruction, the phantom data with a slice outside the object were also reconstructed without slice-GRAPPA. The results for signal changes were then compared. For the DWI data, TrW images were calculated by taking the geometric mean of the three *b* = 1000 s/mm^2^ images acquired with orthogonal diffusion-encoding gradients.

In order to correct for motion-induced differences, all single-contrast data were registered to the corresponding SMC images with a 3D rigid transformation (rotation and translation) based on normalized gradient fields whenever voxel-wise comparison was performed. In all other cases, no registration was performed because the data quality suffers from interpolation during registration, which could have an impact on the comparison results.

To assess the effect of simultaneously acquired contrasts, a comparison between the SMC acquisition and the separate contrast measurements for four subjects (all slices) was performed using the following techniques: (a) Pearson correlation coefficient (PCC) [44], (b) structural similarity index measure (SSIM) [45] for all slices, (c) scatter plots, (d) difference images, and (e) histograms of the masked difference images. SSIM is a well-known quality metric that can be used to measure the similarity between two images. It was developed by Wang et al. [36] and is considered to be correlated with the quality perception of the human visual system. Rather than using conventional error summation methods, the SSIM is modeled by taking each image distortion as a combination of three factors representing loss of correlation, luminance distortion, and contrast distortion. The SSIM is defined as:

$$S \left(x,y\right)=f(l\left(x,y\right),c\left(x,y\right),s\left(x,y\right)),$$

where *l(x,y)* is the luminance comparison function that measures the approximation of the average luminance of the two images, *c(x,y)* is the contrast comparison function that measures the closeness of the contrast of the two images, and *s(x,y)* is the structure comparison function that measures the correlation coefficient between the two images f and g. The positive values of the SSIM index are in the range [0,1]. A value of 0 means no correlation between the images, and 1 means that *f* = *g*. It is important that the three components are relatively independent of each other. Changing the luminance and/or contrast, for example, has no effect on the structures of the images [45].

Results for the two-slice data from inside and outside the head were compared using the following three options for the source of ACS data for the slice-GRAPPA weight calculation: (a) *b*_0_ scan and a T_2_*W scan, (b) *b*_0_ scan only, and (c) T_2_*W scan only. Ideally, the signal above noise level in the positions outside the brain should be zero after signal separation, so the visual inspection of residual signal and entropy in this slice was used to compare the performance of the three options.

## Results

**Fig. 2:**
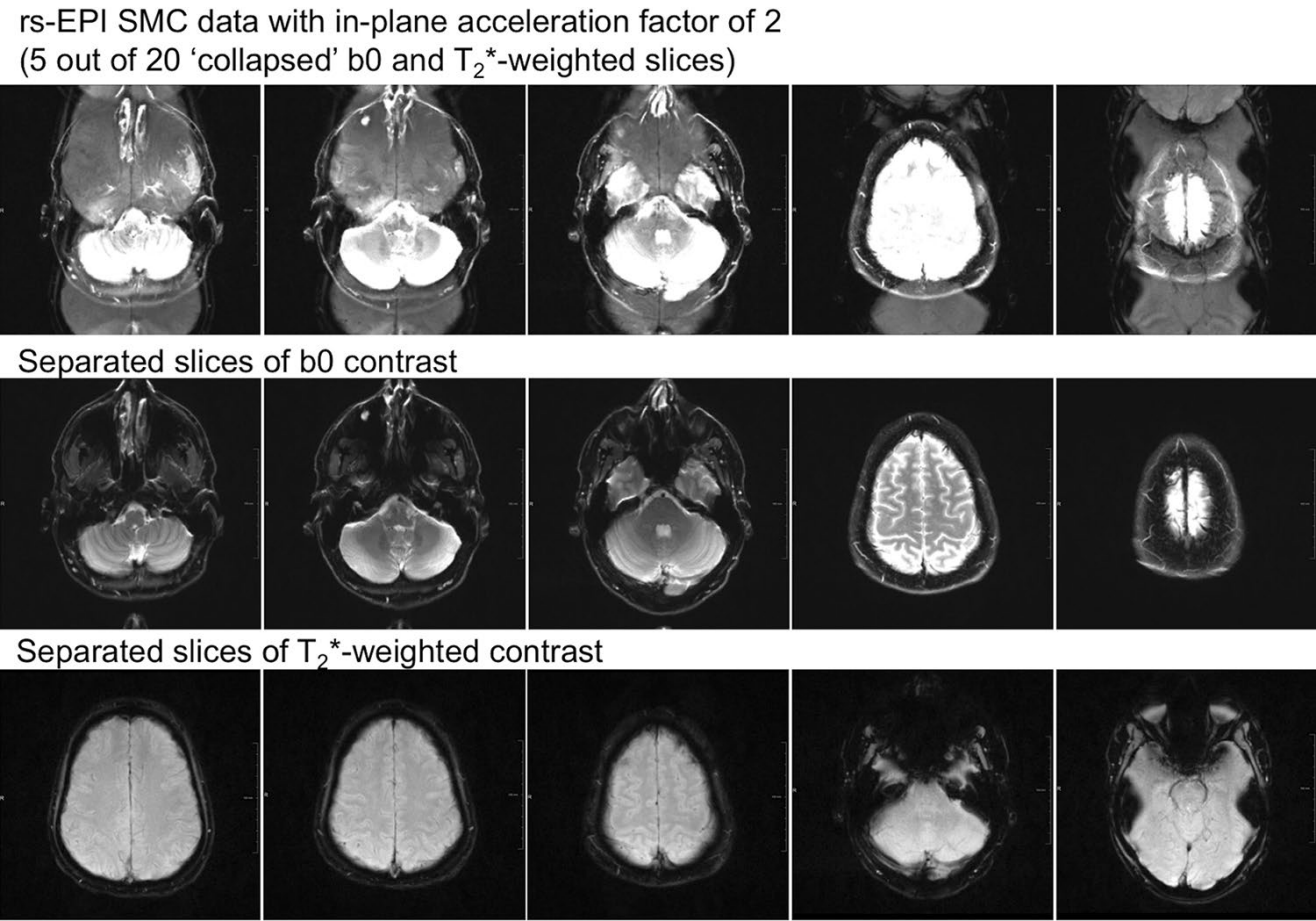
Data from five out of 20 measured SMC slices. The first row shows the collapsed SMC data with an in-plane acceleration factor of 2; The slices from the DW acquisition (with *b* = 0 s/mm^2^) are at the center of the FOV while the T_2_*W slices are shifted by FOV/2 in the anterior–posterior direction. The middle row shows the DWI data and the bottom row shows the T_2_*W data

Figure 2 shows the two collapsed slices from a SMC scan (i.e. without slice separation) at five out of the 20 different positions and the corresponding separated b_0_ and T_2_*W images.

**Fig. 3:**
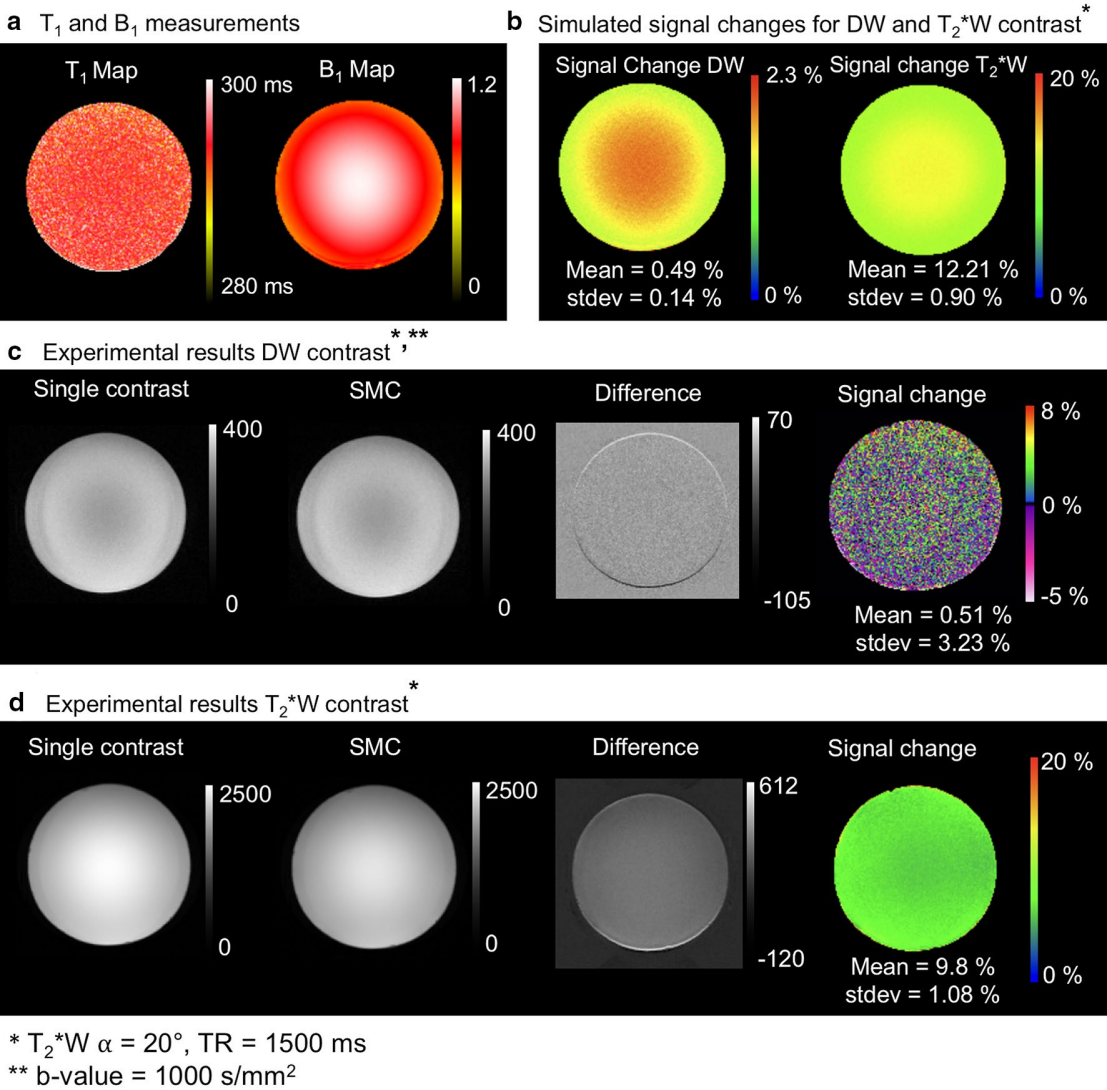
**a** Measured *T*_1_ and *B*_1_ maps in the spherical water-based phantom, **b** Simulated magnetization signal changes [%] with Bloch equations on the basis of the *T*_1_ and *B*_1_ map, **c** DW acquisition without and with simultaneous acquisition of T_2_*W contrast, the corresponding difference image and signal-change map, **d** T_2_*W contrast without and with simultaneous acquisition of the DW contrast, the corresponding difference image and signal-change map. The difference images show evidence of an uncorrected frequency drift during measurement in the outer ring. These areas of high differences are masked out in the saturation maps. Positive values [%] of signal change means smaller grey values in the SMC images than in the single-contrast images and vice versa

Figure 3 shows the results of the analysis of signal-change effects in the phantom. For both contrast types, the Figure compares SMC data with the corresponding single-contrast images to determine experimental signal-change effects (c & d) due to the SMC method. These results are compared with signal-change factors given by the simulations (b). The simulations are based on T_1_ and B_1_ measurements (a). A positive value for the percentage signal difference means that the SMC images have less signal in this region compared to the single-contrast images.

**Fig. 4:**
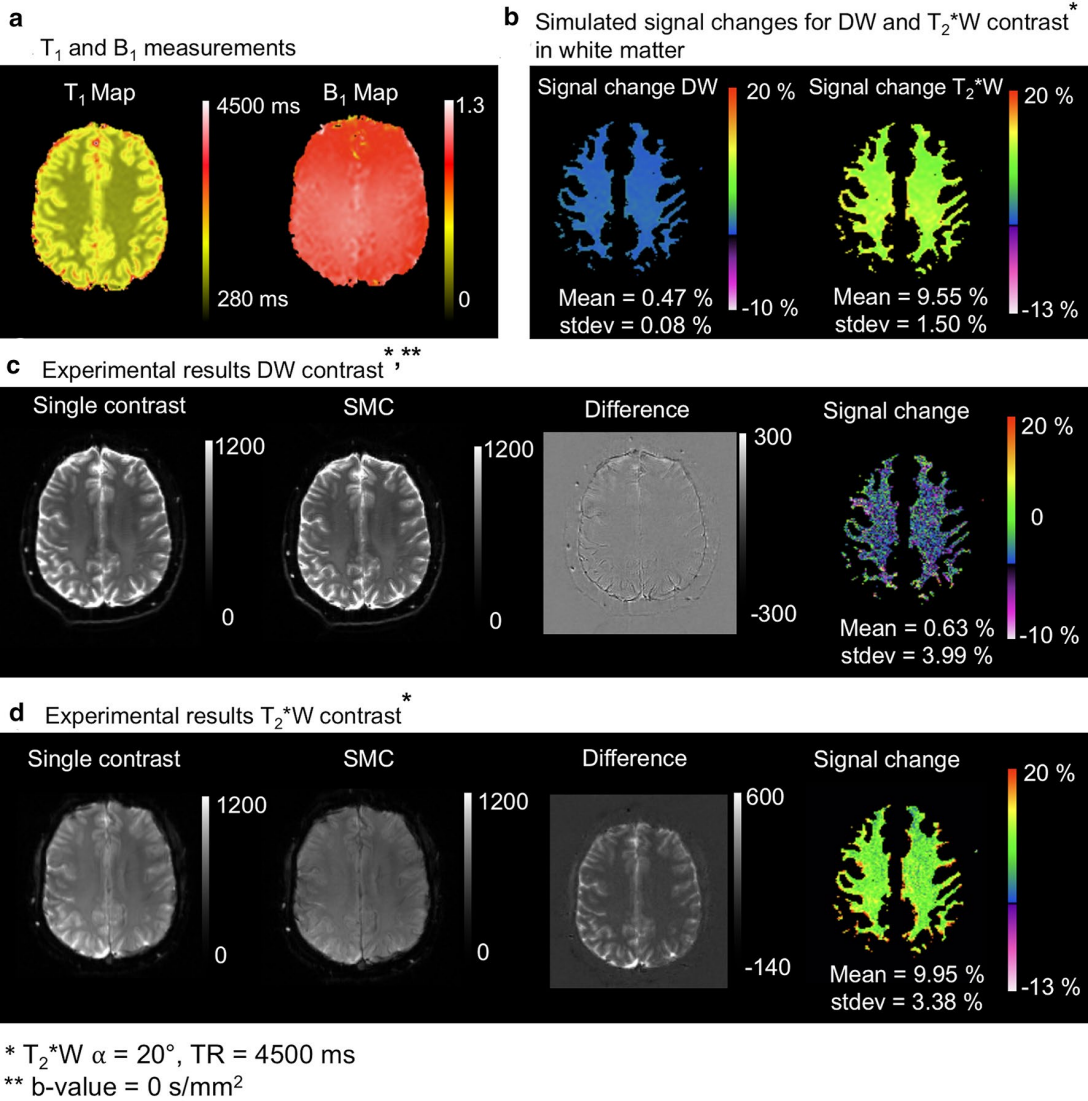
Signal change result for slice iteration scheme A: **a** Measured T_1_ and B_1_ maps in vivo, **b** Simulated magnetization signal change [%] with Bloch equations on the basis of the T_1_ and B_1_ map in white matter, **c** DW acquisition without and with simultaneous acquisition of T_2_*W contrast, the corresponding difference image and signal change map, **d** T_2_*W contrast without and with simultaneous acquisition of the DW contrast, the corresponding difference image and signal change map. Positive values [%] of signal change means smaller grey values in the SMC images than in the single-contrast images and vice versa

**Fig. 5:**
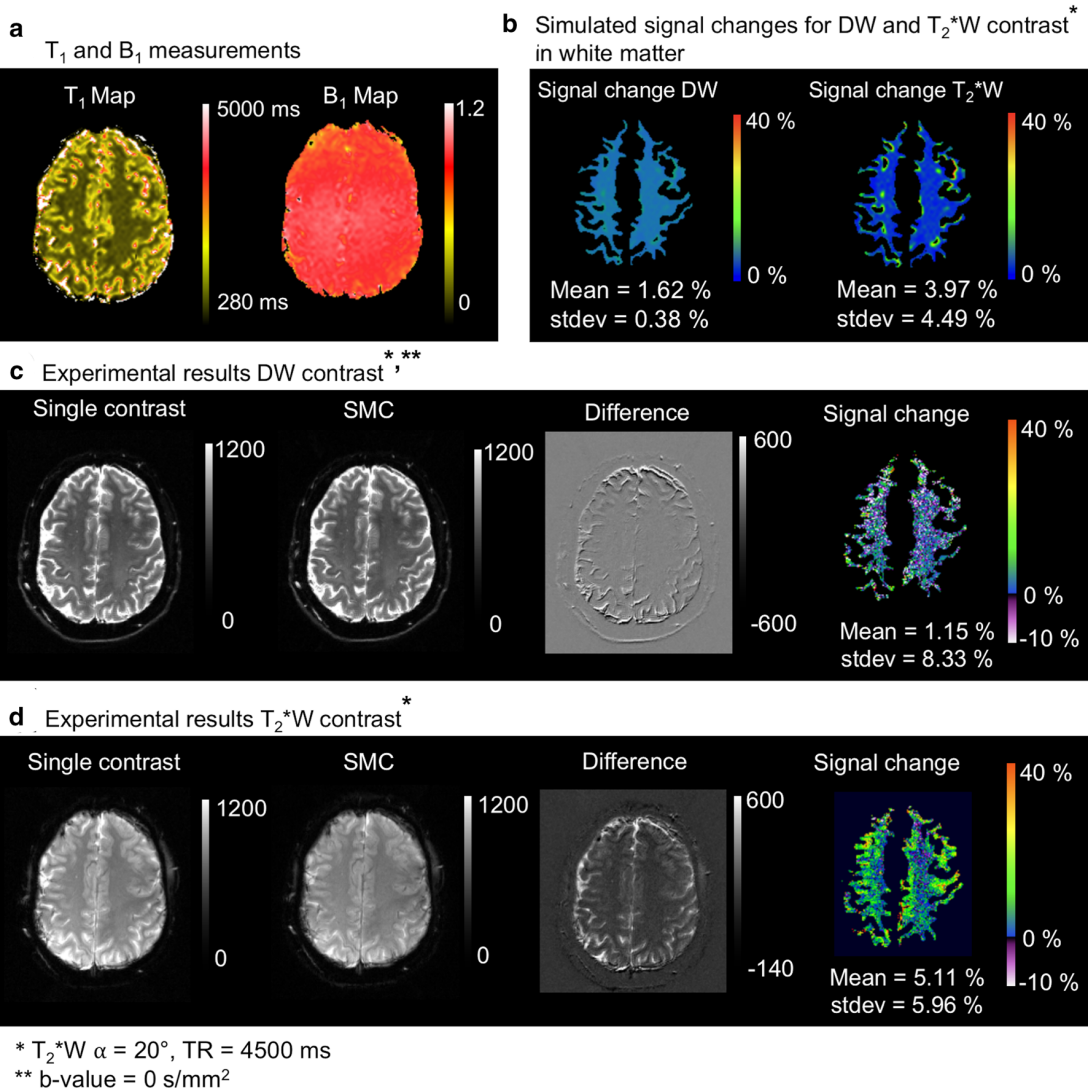
Signal change results for slice iteration scheme B: **a** Measured T_1_ and B_1_ maps in vivo, **b** Simulated magnetization signal change [%] with Bloch equations on the basis of the T_1_ and B_1_ map in white matter, **c** DW acquisition without and with simultaneous acquisition of T_2_*W contrast, the corresponding difference image and signal change map, **d** T_2_*W contrast without and with simultaneous acquisition of the DW contrast, the corresponding difference image and signal change map. Positive values [%] of signal change means smaller grey values in the SMC images than in the single-contrast images and vice versa

The results of the corresponding analysis for signal-change effects in vivo are shown in Figs. 4 and 5. Simulation and signal-change results are only shown for WM (b). Corresponding data for GM are shown as supporting material (Supporting Information Figures S3 & S4). The experimental data in the Figure show a low signal-change factor for DW contrast (c) and a larger signal-change factor for T_2_*W images (d).

**Fig. 6:**
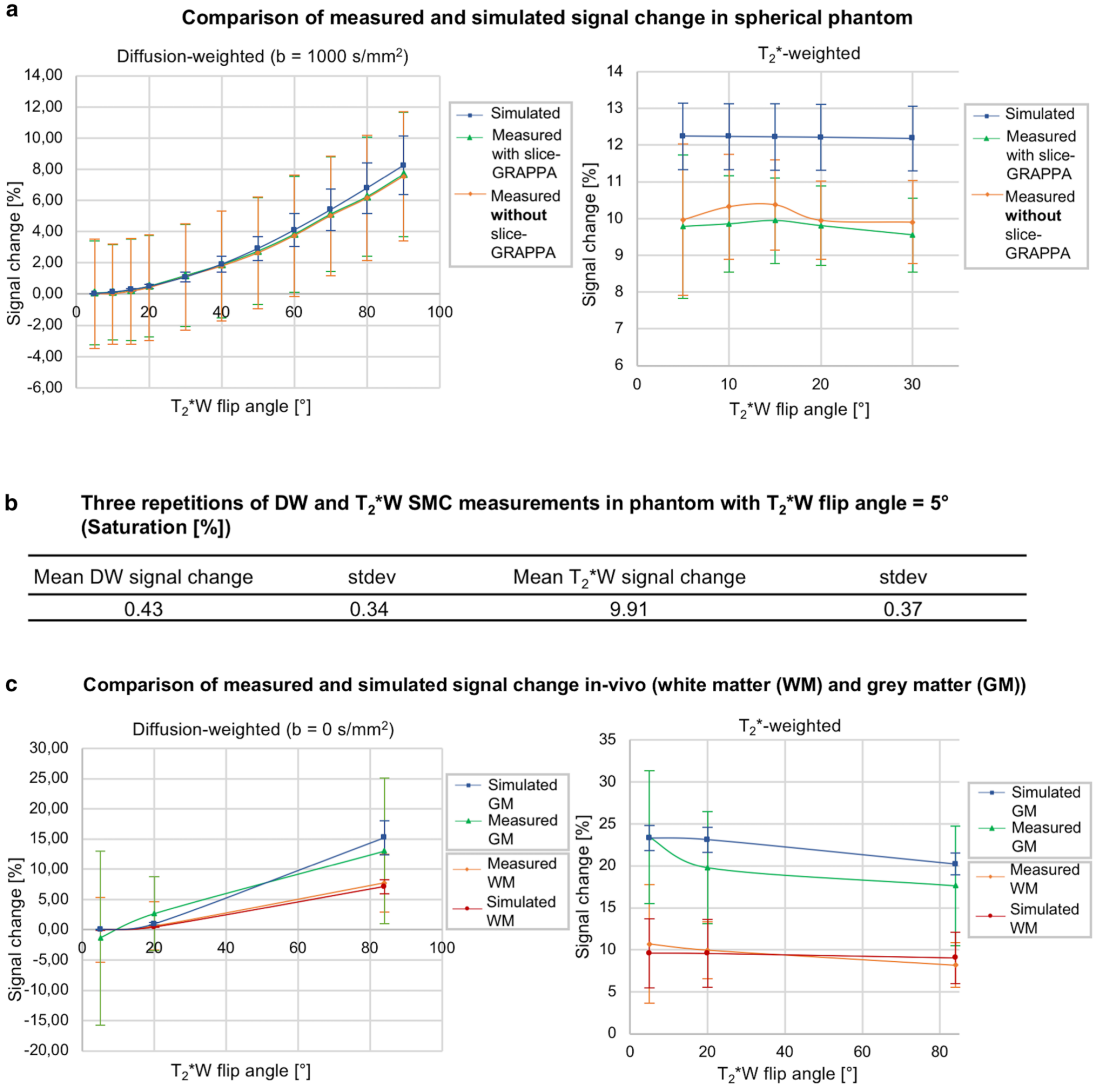
**a** Comparison between simulated and measured signal change with and without split slice-GRAPPA reconstruction in the spherical phantom. **b** The table shows the mean signal change in terms of signal saturation and standard deviations for both contrasts in the phantom between three repeated measurements. **c** Comparison between simulated and measured signal change in vivo in grey and white matter (GM, WM) respectively

Figure 6 shows the results of the simulated and experimentally determined signal-change effects of the two-slice data in the phantom (a) and in vivo for WM and GM (c). In each case, the plots show signal-change factors for DW and T_2_*W imaging for a range of flip angles for the T_2_*W excitation pulse. The mean signal-change factor for the phantom experiment is also shown in the Figure for the case of three repetitions (b).

**Fig. 7:**
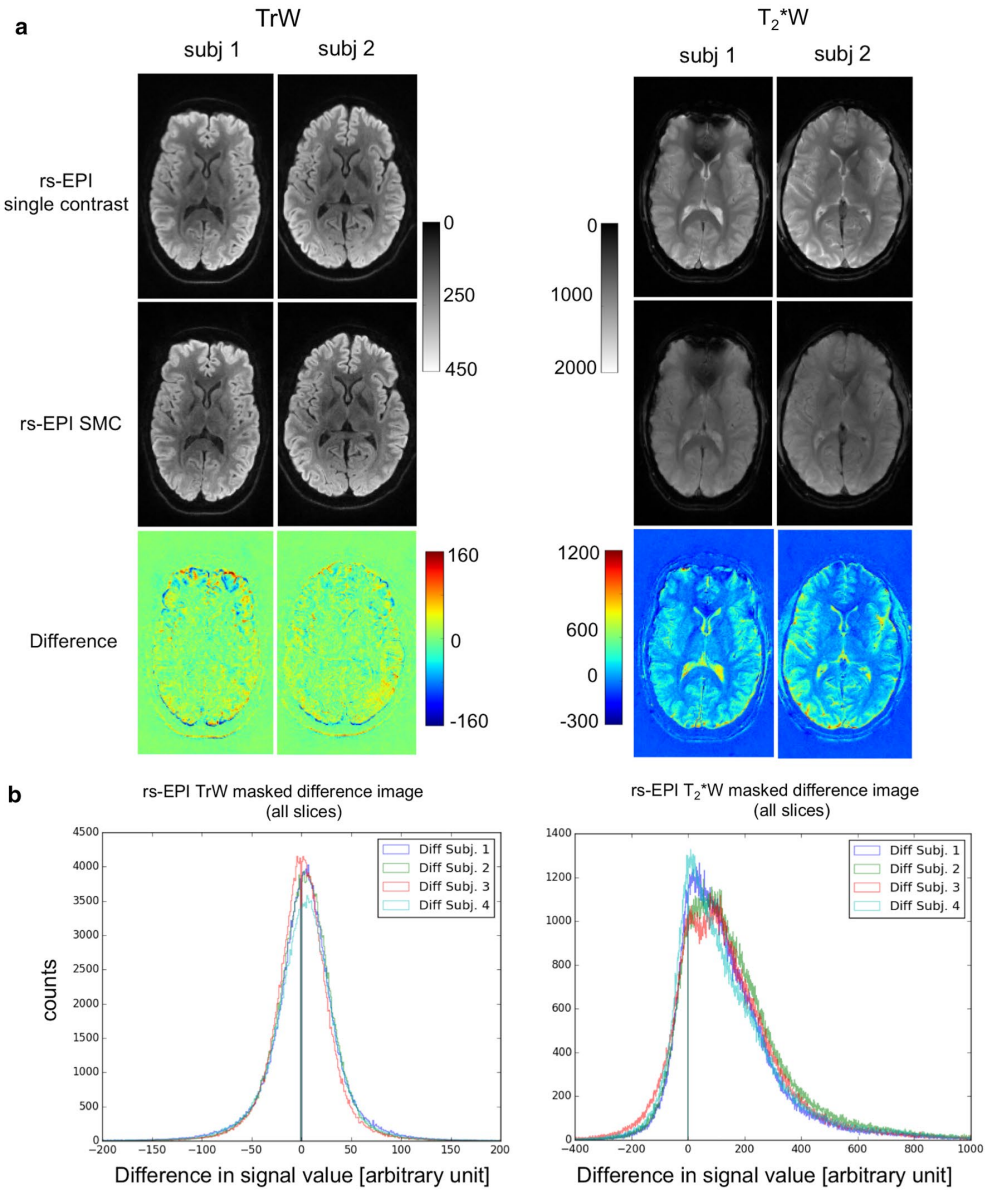
**a** Difference images between rs-EPI single-contrast (DW trace-weighted and T_2_*W) and rs-EPI SMC (separated trace-weighted and T_2_*W contrast) in-vivo central slice out of 20 slices. **b** Histograms of all slices in all subjects of the masked difference image

Figure 7 shows the differences between the single-contrast and SMC images for a central slice in two out of four subjects. On visual inspection, there is little difference between the DW images acquired with SMC and those acquired with the standard single-contrast method. In the T_2_*W images, the SMC images mainly show signal reduction affecting the CSF and GM compared with the single-contrast images. The figure also shows a histogram analysis for the distribution of voxel-to-voxel intensity differences between the SMC and single-contrast data sets for all 20 slices from each of the four subjects. The image data were masked to eliminate contributions from background noise. The skewed distribution for the T_2_*W data indicates the systematic effect of saturation on signal intensity when all tissue types are considered.

**Fig. 8:**
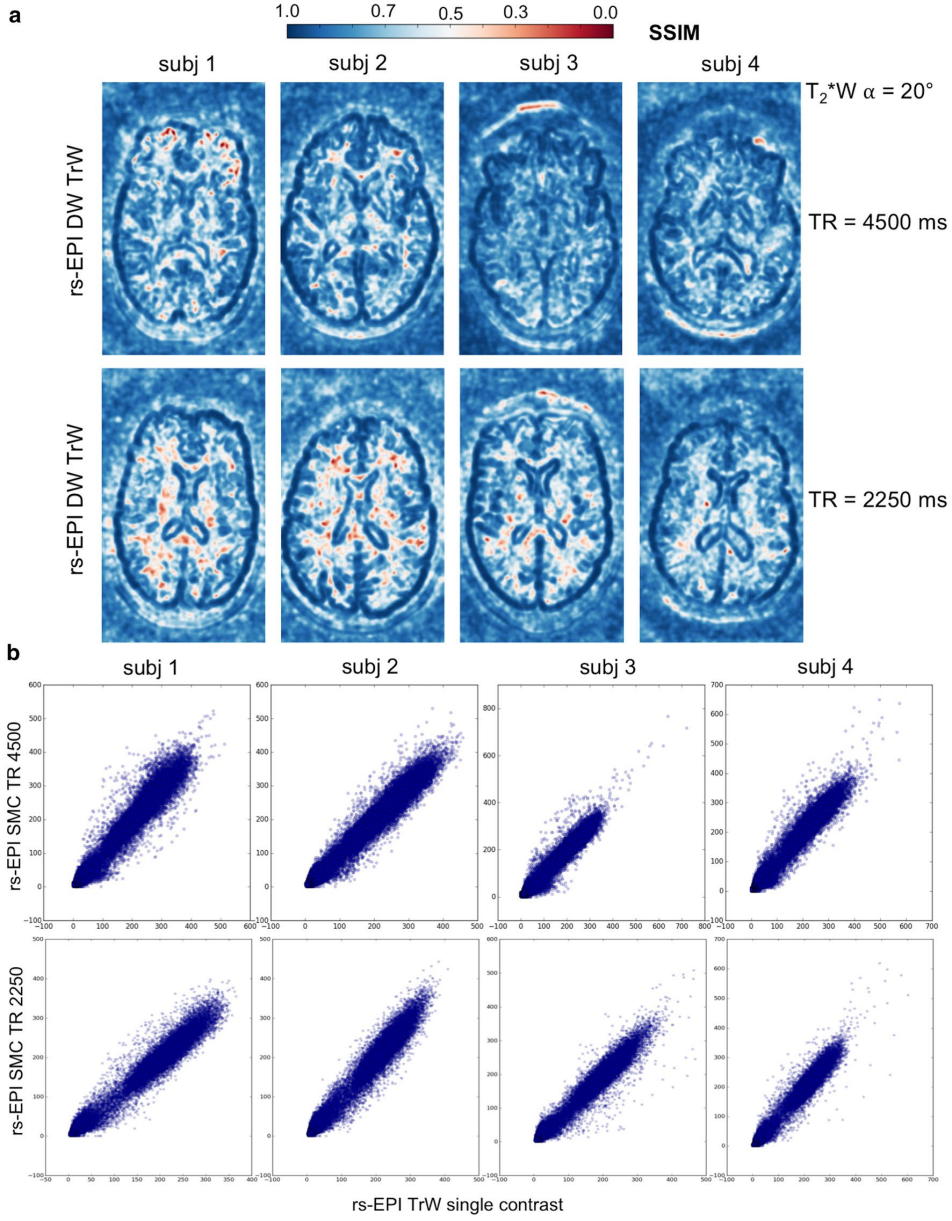
**a** The structural similarity index measure (SSIM) maps of the central slice of the calculated trace-weighted data for TR = 4500 ms and TR = 2250 ms. **b** Respective scatter plots of the gray level distributions of single-contrast and SMC data in the central slice

**Fig. 9:**
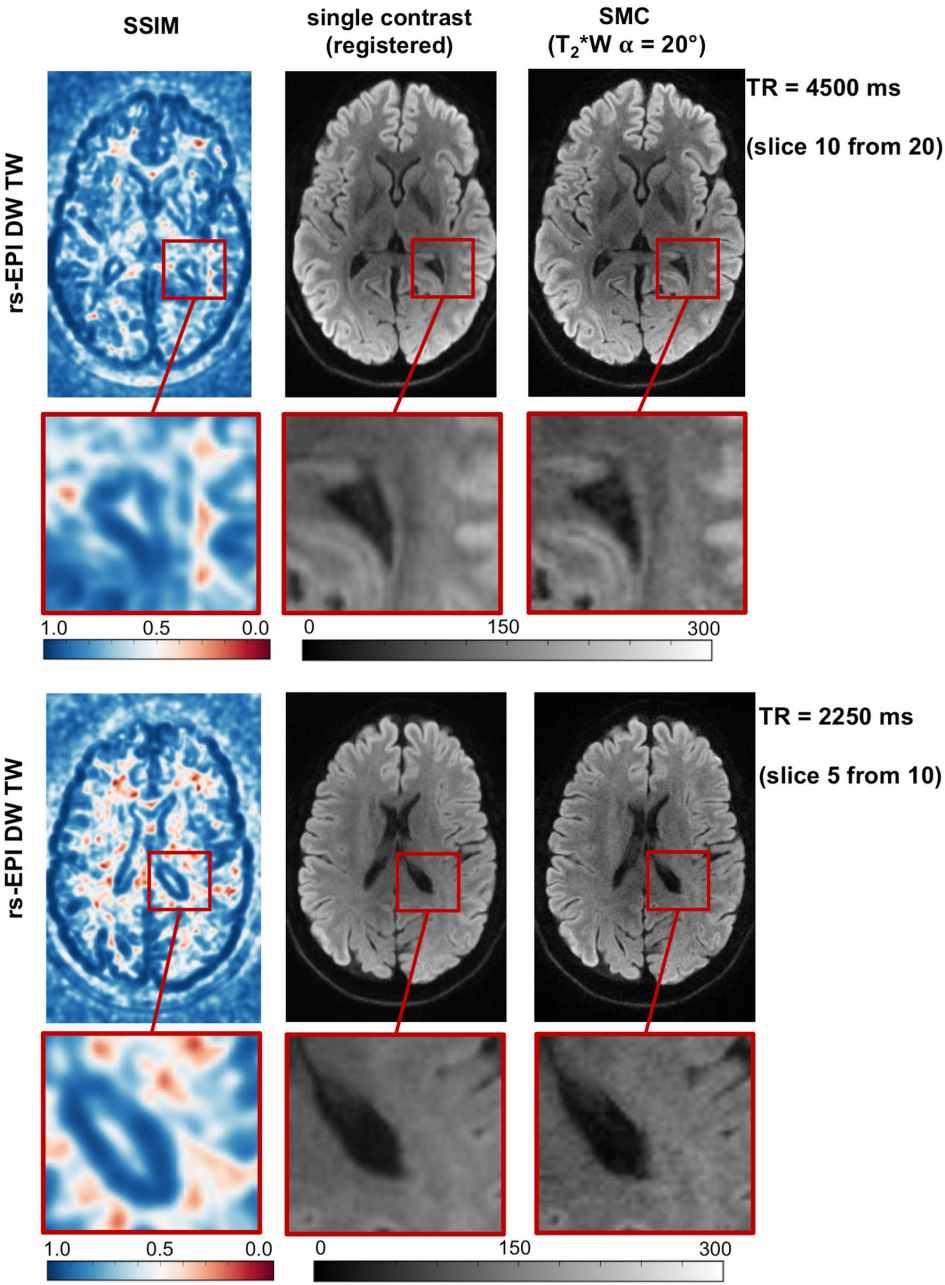
Comparison between the registered TrW single-contrast and central SMC slices of subject 2 with the corresponding SSIM map for TR and TR/2. The close-up image allows better interpretation of the correlation between high and low SSIM values and structural changes between the single-contrast and SMC slices

Figure 8 shows the SSIM maps (a) of the central slice of TrW data for TR = 4500 ms and TR = 2250 ms. For each TR value, these maps provide a measure of the structural similarity (none (0.0) to perfect (1.0)) between images acquired with SMC and images acquired with standard single-contrast imaging. The scans with the shorter TR show an increase in the number of voxels with low structural similarity. The scatter plots (b) show the correspondence between single-contrast and SMC voxel intensities in the central slice. Additionally, Fig. 9 shows for subject 2 the TrW slice with the corresponding SSIM map and a close-up view of both.

Table 2 presents the results of the similarity analysis between single-contrast and SMC data for TrW and T_2_*W images. The respective mean and standard deviation of the PCC and SSIM were computed over all acquired slices (20 for TR = 4500 ms and 10 for TR = 2250 ms). To reduce the influence of background noise, the images were cropped in the readout direction. Voxels outside the head in the phase-encoding direction were included in the analysis to include possible ghosting artifacts. The PCC is in the high range above 0.9 for all slices and subjects without exception. SSIM ranges from 0.0 in a few locations to 1.0. The mean SSIM ranges from a lowest value of 0.68 for T_2_*W contrast (TR = 4500 ms) to a highest value of 0.83 for TrW contrast (TR = 4500 ms). For TrW contrast, SSIM decreased in all subjects when TR was reduced from 4500 to 2250 ms. No similar effect was seen for the T_2_*W contrast.

**Table 2:** Similarity Measurements rs-EPI trace-weighted single-contrast—rs-EPI trace-weighted SMC (T_2_*W $\alpha$ = 20°)^a^

TrW	TR = 4500 ms				TR = 2250 ms			
	PCC		SSIM^b^		PCC		SSIM^b^	
Subject #	mean	stdev	mean	stdev	mean	stdev	mean	stdev
1	0.94	0.03	0.82	0.03	0.97	0.01	0.80	0.03
2	0.97	0.02	0.82	0.03	0.98	0.01	0.77	0.03
3	0.96	0.01	0.80	0.05	0.98	0.01	0.80	0.04
4	0.94	0.01	0.83	0.03	0.97	0.01	0.79	0.03

T_2_*W	TR = 4500 ms				TR = 2250 ms			
	PCC		SSIM^b^		PCC		SSIM^b^	
Subject #	Mean	stdev	Mean	stdev	Mean	stdev	Mean	stdev
1	0.95	0.04	0.74	0.04	0.97	0.01	0.73	0.05
2	0.94	0.05	0.73	0.06	0.97	0,01	0.72	0.05
3	0.92	0.07	0.68	0.07	0.97	0,01	0.72	0.06
4	0.95	0.03	0.70	0.04	0.97	0,01	0.71	0.05

^a^Cropped images in readout direction were used to limit influence of background noise

^b^Registered images were used for analysis

All p-values are < 0.0001, and therefore the measured similarity is significant at p < 0.05

*PCC* Pearson correlation coefficient, *SSIM* structural similarity index measure

**Fig. 10:**
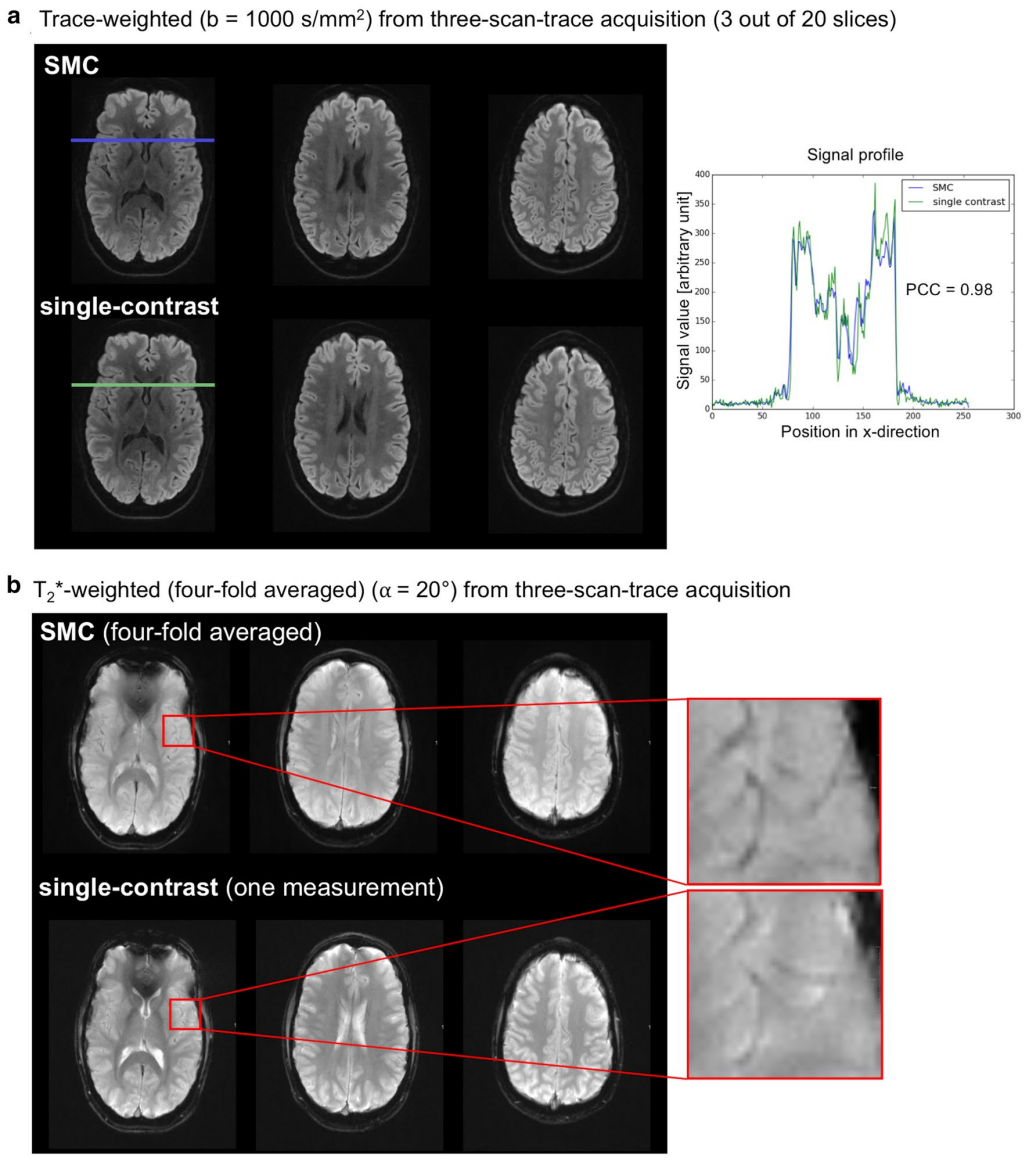
Contrast comparison in the central slice in one of four subjects. **a** Comparison of signal profile between single-contrast and SMC trace-weighted image. **b** Comparison of contrast between veins and tissue in T_2_*W contrast

Figure 10 shows for a single subject, TrW and T_2_*W images from 3 out of the 20 slices. The T_2_*W images shown are the mean of four scans to match the four DW images (one image for *b* = 0 s/mm^2^, and three images for *b* = 1000 s/mm^2^). Images are shown for both SMC and single-contrast data acquisitions for comparison. For the TrW data, a profile plot through all tissue types shows good agreement between SMC and single-contrast acquisitions. The PCC between the two profiles is 0.98. For the T_2_*W data, the zoomed images show that vessel detail can be seen clearly in both scans, despite the signal-change effect on the SMC images.

**Fig. 11:**
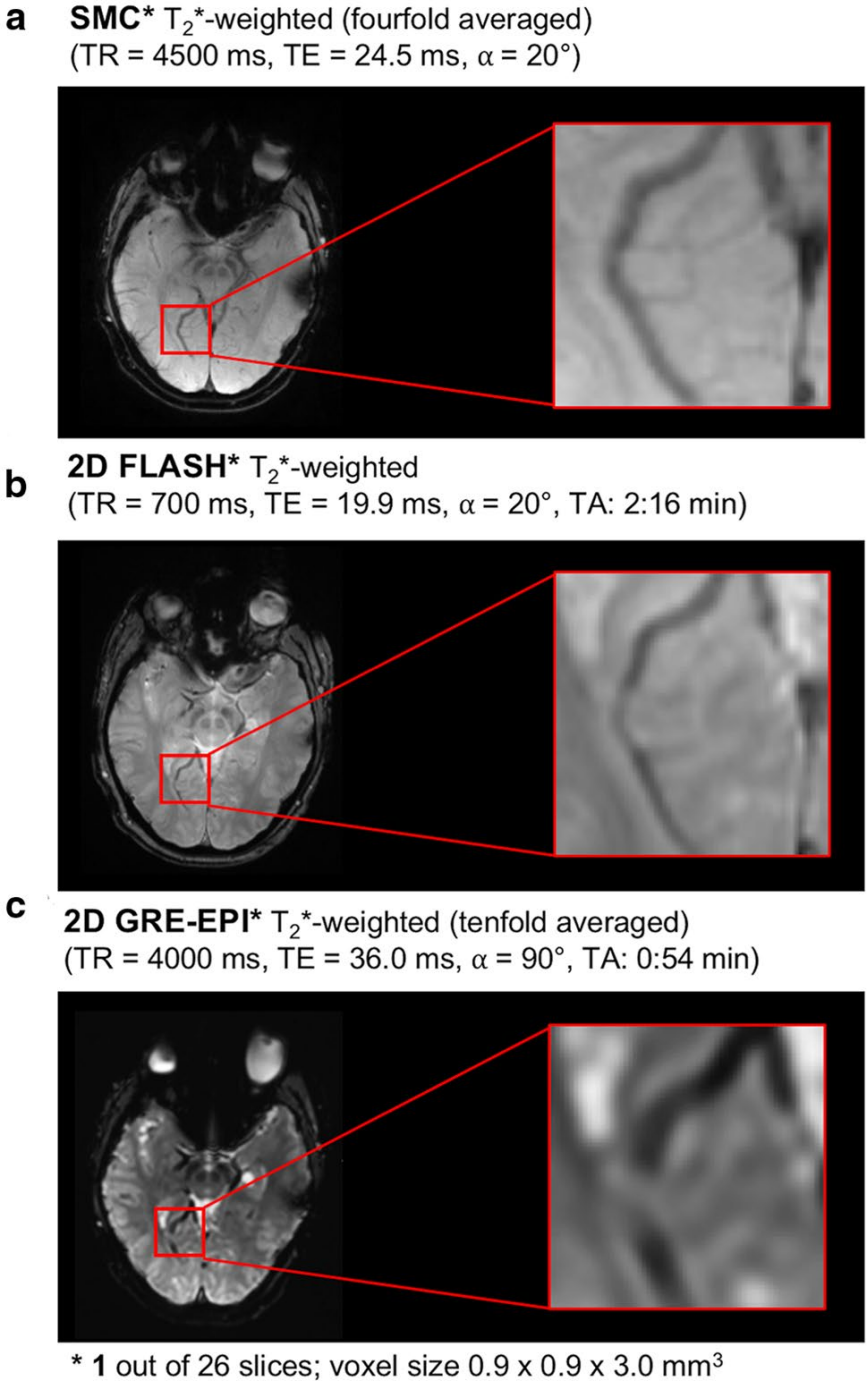
Comparison between an T_2_*-weighted SMC, 2D Flash and GRE-EPI (both product) measurement in terms of scan time and visual vein-tissue contrast

Finally, the vein-tissue contrast of the T_2_*W SMC images from 1 out of 26 slices is compared with the results of 2D FLASH and 2D GRE EPI standard sequences with different measurement times and image contrasts in Fig. 11.

Results for the comparison between slice separation using different sources of ACS data are shown as supporting material (Supporting Information Figure S5 and Supporting Information Table S1). All three ACS options (*b* = 0 s/mm^2^ only, T_2_*W only, and one of each for the respective contrast type) provided good separation. Best results were, however, achieved using a *b* = 0 s/mm^2^ ACS scan only, which has the additional benefit of reducing the number of reference scans that are required at the start of the measurement.

## Discussion

The SMC imaging technique described in this study allows a combination of contrasts within a single MRI pulse sequence. The results demonstrate that the method can be used to incorporate an additional T_2_*W contrast into a DW rs-EPI sequence to give good quality images for both contrast types for a scan time that is similar to the original single-contrast DW scan.

### Bloch simulation and experimental magnetization signal-change effects

One challenge to the integration of a second contrast into an MRI pulse sequence is the confounding effect of signal-change effects. To optimize tradeoff between SNR in the DW and T_2_*W images, and scan time, Bloch simulations and experimental data in phantom and in vivo were considered (Figs. 3–6). In general, there was good agreement between simulated and experimental data, but there were some discrepancies, in particular, the overestimated signal-change for T_2_*W contrast in phantom and in GM (in-vivo) given by the simulation. This might be due to the effects of slice profile, which was not accounted for in the simulation [23]. The relatively high standard deviations of the mean signal-change (error bars in Fig. 6) could be due to normal differences between individual measurements (Fig. 6b & S2) and imperfect registration in vivo. Signal-change effects limit the types of contrast that can be efficiently combined using SMC. This will need to be carefully considered in future work, with Bloch simulations playing an important role in identifying the best SMC acquisition schemes.

In the current implementation, the effect of signal change is reduced by using a low flip angle for the T_2_*W acquisition. With this approach, the additional T_2_*W acquisition has a minimal signal-change effect on the DW signal in slice iteration scheme A, whilst achieving a good SNR for the T_2_*W images, which were acquired with four averages. Differences between single-contrast TrW and SMC-TrW images appear to result primarily from residual position differences due to imperfect registration between the single- and multiple-contrast scans (Fig. 7a). One reason for imperfect 3D registration could be the relatively large gaps between the slices. In general, the effect of saturation on the measured signal is at a similar level as the signal fluctuations due to noise in the DW images.

A more significant impact of signal change is seen when examining the effect of the DW acquisitions on the T_2_*W signals. This can be seen in the asymmetric histogram distribution for the difference between T_2_*W pixel values in the single-contrast and SMC scans (Fig. 7b). A particularly high signal-change effect is seen in CSF, where T_1_ times are longer than in GM and WM. However, the SNR loss in the T_2_*W images is mitigated by acquiring four averages corresponding to the four DW scans (*b* = 0 s/mm^2^ and 3 × *b* = 1000 s/mm^2^, compare Fig. 10b). Four-fold averaging increases the total image SNR by a factor of 2, while SNR loss due to saturation is 20% for GM and 10% for WM.

Within certain limits, the choice of the slice iteration scheme has an influence on the mutual influence of the two excitations. For example, slice iteration scheme B leads to a lower influence on the T_2_*W scans and increases the influence on the DW scans by only a few percentage points. Therefore, depending on the contrast combinations and desired application, it might be beneficial to use a different slice iteration scheme and determine which flip angle and TR is the best choice in this case. In this work, DW contrast was prioritized over T_2_*W contrast because the vein-tissue contrast should be adequate even at higher signal saturation (compare Figs. 10 & 11). Other slice iteration schemes can be used to achieve a different balance between the signal-change effects affecting the different contrasts. To illustrate this point, a comparison between three different slice iteration schemes is provided in the supporting material (Supporting Information Figure S6).

### Similarity measurements and image contrast considerations

Single-contrast TrW and T_2_*W images and the corresponding SMC data show high similarity at both TR values used in the study. As shown in Table 2, the PCC shows high similarity with a low standard deviation across all slices. However, PCC is not ideal for detecting structural changes and saturation effects. SSIM considers texture, contrast and luminance [45] and is therefore, better suited. Due to the small flip angle, the T_2_*W contrast is less sensitive to TR reduction than DWI, whose values are reduced when the shorter TR is used (Table 2a).

The visual difference between the TrW single-contrast and SMC data in Fig. 9 is partly due to the registration process. Due to the interpolation performed, the single-contrast data looks rather blurry. However, this step was necessary to perform a voxel-by-voxel comparison with SSIM. Structural changes indicated by high SSIM values could be detected by a more detailed examination of the image details.

The overall image contrast in TrW images can be further analyzed by considering the signal differences between air, GM, WM, and CSF. The signal profile in Fig. 10a shows a high similarity between single-contrast and SMC images, demonstrating further that the low-flip-angle T_2_*W acquisition could be added to the sequence without a major influence on the DW images.

For clinical application, it is important that the signal change effects in SMC do not result in contrast changes that might limit the detection of hemorrhage in the T_2_*W images. The overall image contrast of T_2_*W-SMC images is reduced compared to the single-contrast scans mainly due to high saturation in CSF regions (Fig. 10b). Although this has a clear visual effect on the appearance of the image, CSF signal does not have a critical role in identifying and characterizing hemorrhage in the brain. In fact, standard 3D protocols for T_2_*W imaging, such as those used for susceptibility-weighed imaging (SWI), also show a suppressed CSF signal [46].

As seen in the zoomed images (Fig. 10b), the signal in other tissues is less affected and the veins are still clearly visible. This remains the case even when TR is further reduced, for example, if SMC were combined with SMS acceleration. When TR is reduced, saturation increases relatively uniformly for each tissue type (Supporting Information Figure S6). Visually, veins show a lower signal than the other tissue types even at TR/2 (Supporting Information Figure S7).

The vein-tissue contrast is an important consideration when comparing different T_2_*W stroke protocols. For this reason, the contrast provided by the T_2_*W-SMC images, was compared with images acquired with standard 2D FLASH and GRE-EPI sequences (Fig. 11). The veins are clearly visible in both the SMC and the 2D FLASH measurements, whereas the contrast drops in the GRE-EPI measurement.

### Scan time considerations

The initial combination of TrW and T_2_*W contrasts described in this work is of potential value in the assessment of acute stroke because it allows the acquisition of high-resolution TrW and T_2_*W images with a reduction in overall examination time.

In this study, the measurement time was increased by about 14 s compared to a standard DWI measurement, which is longer than necessary. This is due to: the longer TE caused by the additional T_2_*W excitation pulse, a separate ACS data set for each contrast, and the two preparation measurements. This resulted in a total scan time of 3:09 min. Theoretically, it should be possible for the rs-EPI SMC scan to be performed without the additional ACS and preparation scans, leaving only a small increase in scan time due to the longer TE time. This would involve using the ACS in-plane acceleration data to calculate the weights for separating the collapsed multi-slice contrast signals, which would also eliminate the need for additional preparation scans. Previous work suggests that this is an option because, for SMS with low acceleration factors, the fitted slice-GRAPPA kernels show a clear dependence on the coil sensitivity profiles and not on the training data image contrast [5, 23, 29, 40]. The current study has shown that for separation of SMC data with two different contrasts, separation with ACS data with a single-contrast is possible (Fig. S5, Tab. S1).

The scan-time saving that can potentially be achieved using the SMC technique, compared to the standard case of separate single-contrast scans, depends on a number of factors, assumptions, and contrast combinations. Time savings are therefore very difficult to determine in a generalized way. There are different stroke protocols and requirements that theoretically should be considered. The following scan-time comparison is based on a specific case, in which the standalone single-contrast T_2_*W scan provides the same GM SNR as the four-fold averaged SMC T_2_*W images. For the rs-EPI single-contrast T_2_*W acquisition, the minimum TR (TR_min_ = 1100 ms) was chosen as the basis for calculation. A TR of 4500 ms was chosen to calculate the measurement times of the rs-EPI DW single-contrast and SMC images, as in the experimental protocols used in this study. The SNR of the SMC images is reduced by the effects of signal saturation and noise amplification due to g-factor effects (compare Supporting Information Figure S8). When all these factors are taken into account, a two-fold averaging of the single-contrast T_2_*W images would theoretically be necessary to achieve a similar SNR as the inherent four-fold averaging of the SMC T_2_*W images. For these calculations, the simulated and measured signal attenuation of the SMC T_2_*W in GM was about 20% and the g-factor was 1.2. Based on this analysis, for the contrast combination used in this study, it can be estimated that the SMC scan provides an overall time savings of approximately 13% compared to acquiring separate scans. It should be noted that ss-EPI T_2_*W contrast could be acquired much faster as a separate scan, but at the cost of a reduced image resolution and quality compared to the rs-EPI sequence. A high-resolution 2D FLASH measurement, in contrast, would take correspondingly longer.

An additional advantage of SMC imaging is the inherent co-registration between the two contrasts. However, short-term motion during the measurement may still limit perfect co-registration, because of the differing slice acquisition order for the different contrasts. As mentioned above, for the current study, the time between excitation of one contrast and the subsequent excitation of the other contrast at the same slice position is about half the repetition time. Potential object motion during the acquisition could be addressed by combining SMC with image-based prospective motion correction [47]. In this way, the SMC approach could facilitate an accurate image registration that could be helpful in the detailed characterization of pathology and fine anatomical structures.

Although SMS is a powerful technique for lowering scan times by reducing TR, in clinical protocols, where a relatively small number of slices are acquired, the initial TR cannot be shortened without compromising image quality by losing SNR and image contrast. In these scenarios, SMC could provide an alternative in which the overall examination time, rather than the time for an individual scan, is reduced. TR limitations in SMS are particularly severe where high slice-acceleration factors are possible, such as at higher field strengths and for RF coils with a large number of receive elements. In DWI, high acceleration factors with SMS also require high peak voltages for the refocusing pulse, causing high power deposition, particularly at higher field strengths [46]. In this case, SMC could be used as not only an alternative approach, but also as a complementary method, in which SMS with a low acceleration factor is combined with the SMC method.

Since a reduction in TR leads to higher signal-change effects, this study also analyzed total image contrast and similarity for a possible combination of SMC and an SMS acceleration factor of two by comparing single-contrast and SMC data with half the TR. The results suggest that SMC could easily be combined with low acceleration factors of SMS (Table 2, Figs. 8 & S7). However, a more quantitative analysis should be performed to compare SNR and contrast loss for combined SMC and SMS.

### Further optimizations and future applications

Further work is required to address potential motion-induced signal losses in the DW images because the current implementation of the sequence does not use a re-acquisition scheme to avoid non-correctable, severe phase errors, as described in previous studies using DW rs-EPI [22].

The TE of DWI in this study was also prolonged by factors that were not directly related to the SMC method. Long RF pulse durations were used to allow the gradient reversal method [39] to be used to suppress fat signals in the DW acquisition. In addition, long-duration water-excitation RF pulses were used for the T_2_*W excitation. Standard frequency-selective fat suppression, applied before the DW slice excitation, would remove the need for both of these techniques and shorter RF pulses could be used to reduce the TE for the DW images.

The focus of this proof-of-principle study was to analyze the impact that the SMC method has on signal and image contrast. The protocols chosen to investigate these effects had relatively few slices with large slice spacing, leading to a somewhat coarse coverage. For a clinical protocol based on rs-EPI-DWI, a slice coverage of 26–30 slices with slice thicknesses of 1.5–5 mm and a slice spacing of around 0.6 mm would be desirable [29]. Future work must therefore aim to perform SMC imaging with more slices and a smaller slice spacing. The increased number of slices would require an increase in TR and a possible change in the optimum slice iteration scheme. Alternatively, SMC could be combined with standard SMS to avoid an increase in TR and provide saturation effects which are similar to those seen in the current study.

In the current implementation, both contrasts are acquired with the same readout segments. Further optimization could focus on acquiring different readout segments for DW and T_2_*W scans. This would not only offer the possibility to increase the *k*-space coverage in the readout direction in the T_2_*W case, but could also lead to improved signal separation in the multi-contrast reconstruction, since the two slices would have different spectral content.

The SMC scan protocol used in this study was selected for potential application to imaging in acute stroke, so it was important to keep the scan time short and to acquire TrW data with a small number of diffusion-gradient directions. There might be additional applications, particularly in neuroscience, in which the scan time could be extended to acquire data for a larger number of diffusion-gradient directions, whilst a time series of T_2_*W images are acquired simultaneously for functional magnetic resonance imaging (fMRI). Another possibility is that SMC could be used to perform DW fMRI and BOLD fMRI [32] in a single experiment. For the combination of DW and T_2_*W imaging explored in this study, a further possibility is that the individual T_2_*W images could be used to correct the dynamic distortion in the corresponding diffusion-weighted images. This distortion is generated by eddy currents linked to the applied diffusion gradient and varies with its amplitude and direction. A correction based on T_2_*W image registration is likely to be more robust than the direct registration of DW images due to their varying contrast, particularly at high *b*-value.

Future work will also explore the incorporation of other image contrast types into SMC and the combination with low SMS acceleration factors. For example, the simultaneous acquisition of T_1_- and T_2_-weighted images could be achieved by an appropriate choice of slice excitation order.

Finally, we note that the SMC method presented in this paper is not the only way to incorporate a T_2_*W acquisition into a DW pulse sequence. For example, previous work has exploited the redundant navigator echo after the *b* = 0 scan to acquire an additional T_2_W or T_2_*W image [49]. Another possible alternative would be to use standard SMS acceleration to acquire DW images in the first half of the TR time period and T_2_*W images during the second half of the TR. This approach would provide the same relative timing between the acquisition of the two contrasts as with the SMC method. This alternative approach would have the advantage of allowing different readout types for the two contrasts, but would lose the benefit of the matched eddy-current based distortion between the contrasts that is a property of the proposed SMC method.

## Conclusion

This work has shown that techniques from SMS can be used to sample high-quality DW and T_2_*W images simultaneously with good separation between their respective signals. The new SMC method provides an alternative approach to reducing examination time in clinical studies when SMS is limited by TR constraints. It is a complementary technique that could be combined with SMS to exploit the advantages of both methods. In addition, the method provides DW and T_2_*W images that are inherently co-registered with respect to both subject motion and residual distortions caused by the EPI readout. Finally, SMC is compatible with in-plane parallel imaging, allowing the simultaneous acquisition of EPI-based DW and T_2_*W images with reduced spatial distortion.

## Supplementary Information

Below is the link to the electronic supplementary material.

Supplementary file1 (DOCX 25575 KB)
