# Supplementary material for: Combined acquisition of diffusion and T2*-weighted measurements using simultaneous multi-contrast magnetic resonance imaging
Source: MAGMA. 2021 Dec 2;35(3):421–40. doi: 10.1007/s10334-021-00976-3 (PMC9188537; doi:10.1007/s10334-021-00976-3)

Figure S1:


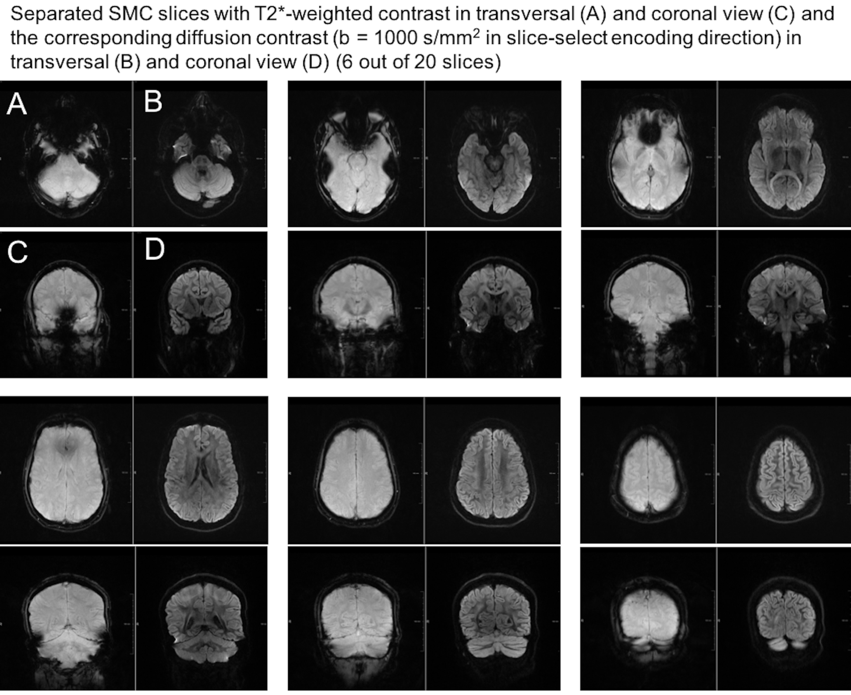


Figure S2:


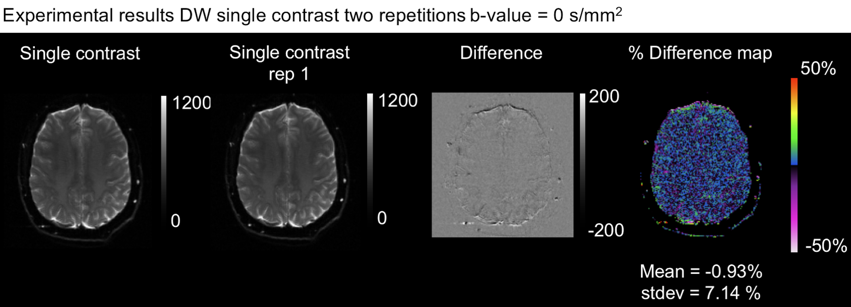


Figure S3: Slice Iteration Scheme A (temp. distance DW 🡪 T_2_*W excitation $\sim\frac{1}{2}\mathrm{TR}$)


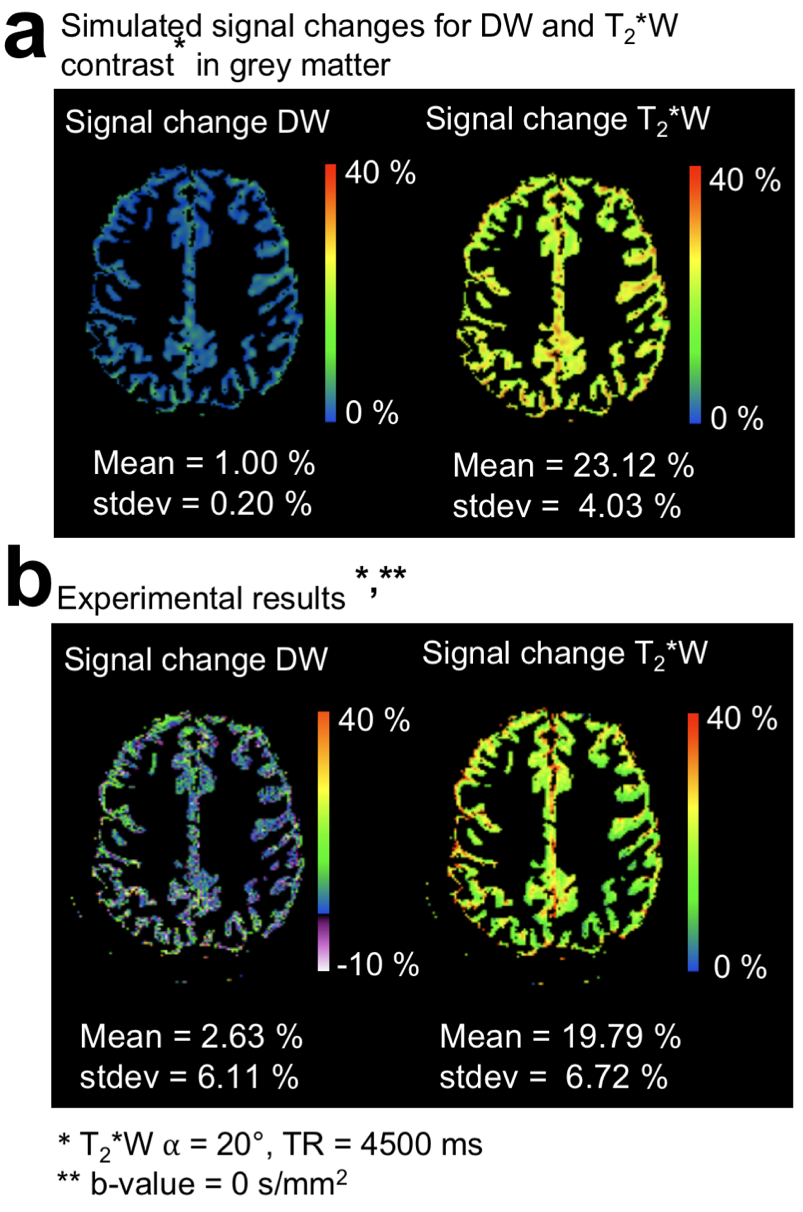


Figure S4: Slice Iteration Scheme B (temp. distance DW 🡪 T_2_*W excitation $\sim\frac{3}{4}\mathrm{TR}$)


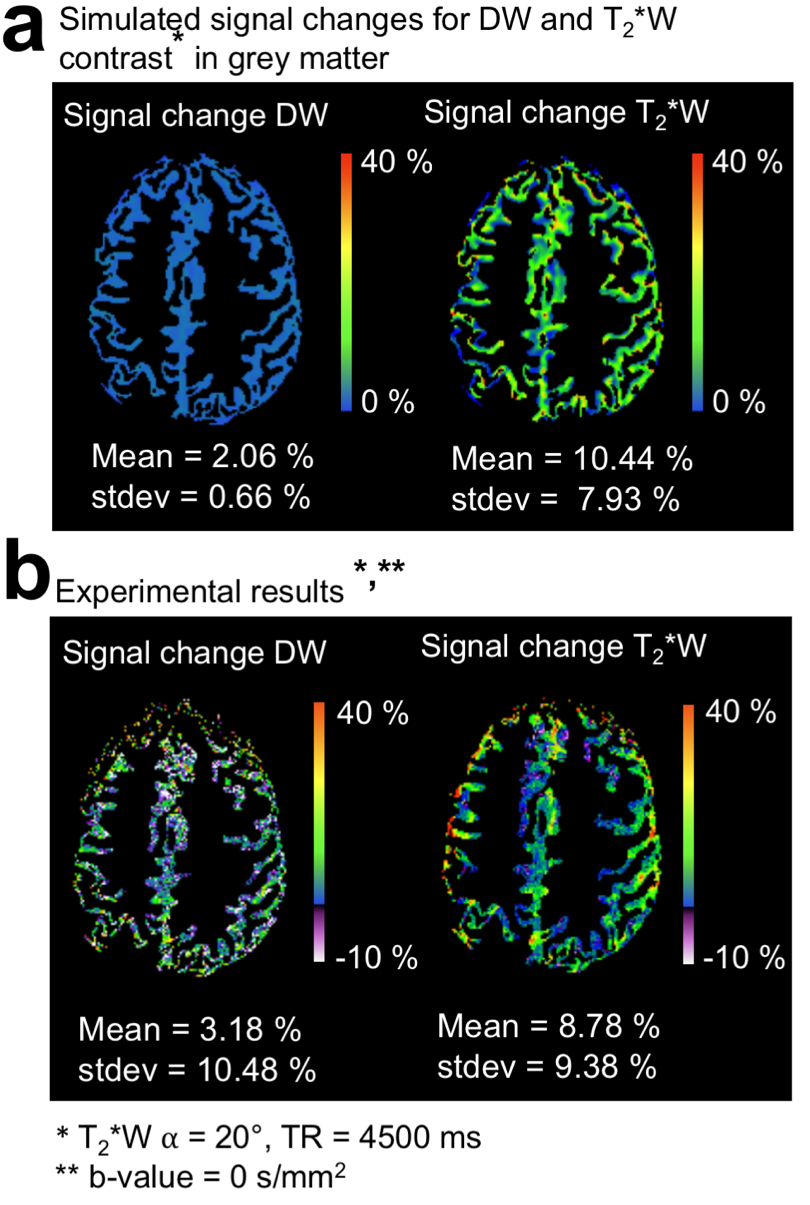


Figure S5:


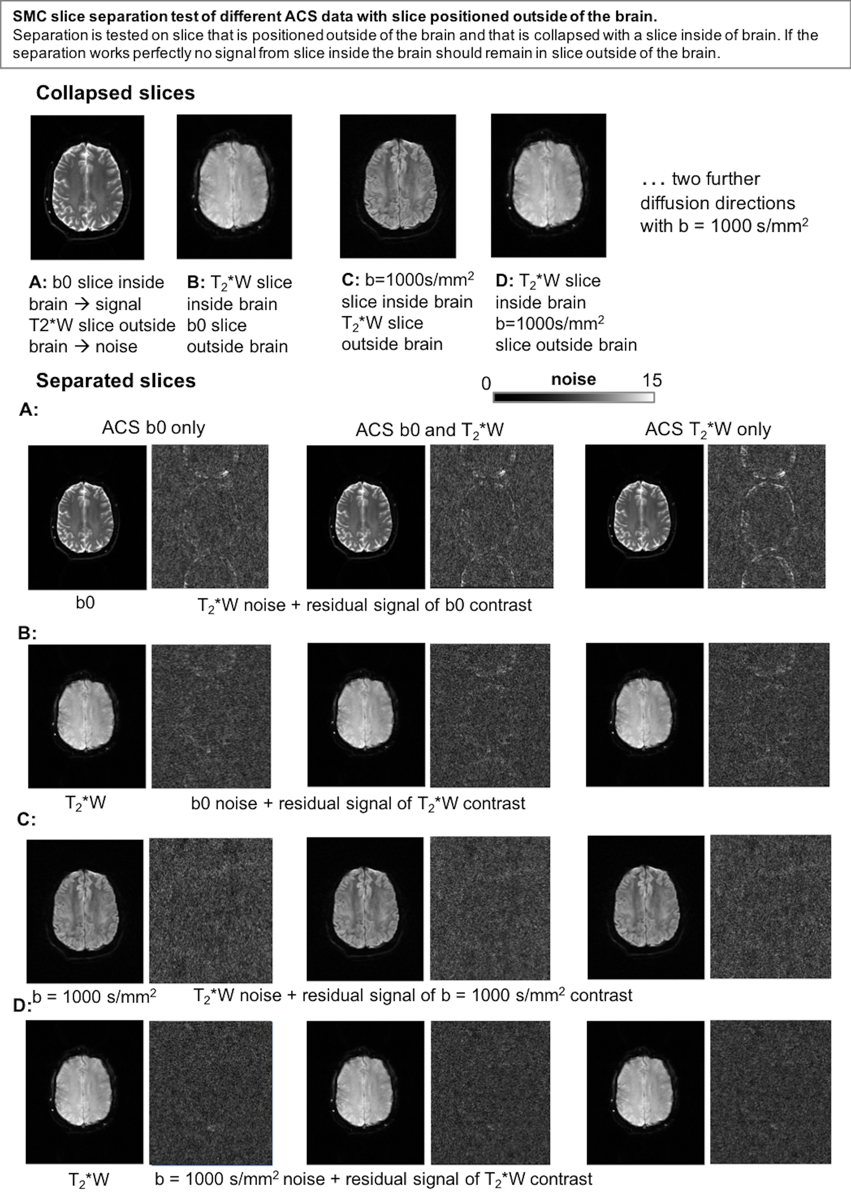


**Table S1:** Residual signal histogram analysis of separated slice outside of head

| **ACS data** | **b0 only** | **b0 and T_2_*W** | **T_2_*W only** |
| --- | --- | --- | --- |
|  | entropy | entropy | entropy |
| **A:**T_2_*W noise  + residual signal of b0 contrast | 2.22 | 2.22 | 2.23 |
| **B:** b0 noise  + residual signal of T_2_*W contrast | 2.11 | 2.08 | 2.09 |
| **C:** T_2_*W noise  + residual signal of b = 1000 s/mm^2^ contrast | 2.18 | 2.18 | 2.17 |
| **D:** b = 1000 s/mm^2^ noise  + residual signal of T_2_*W contrast | 2.03 | 2.01 | 2.01 |

Figure S6:


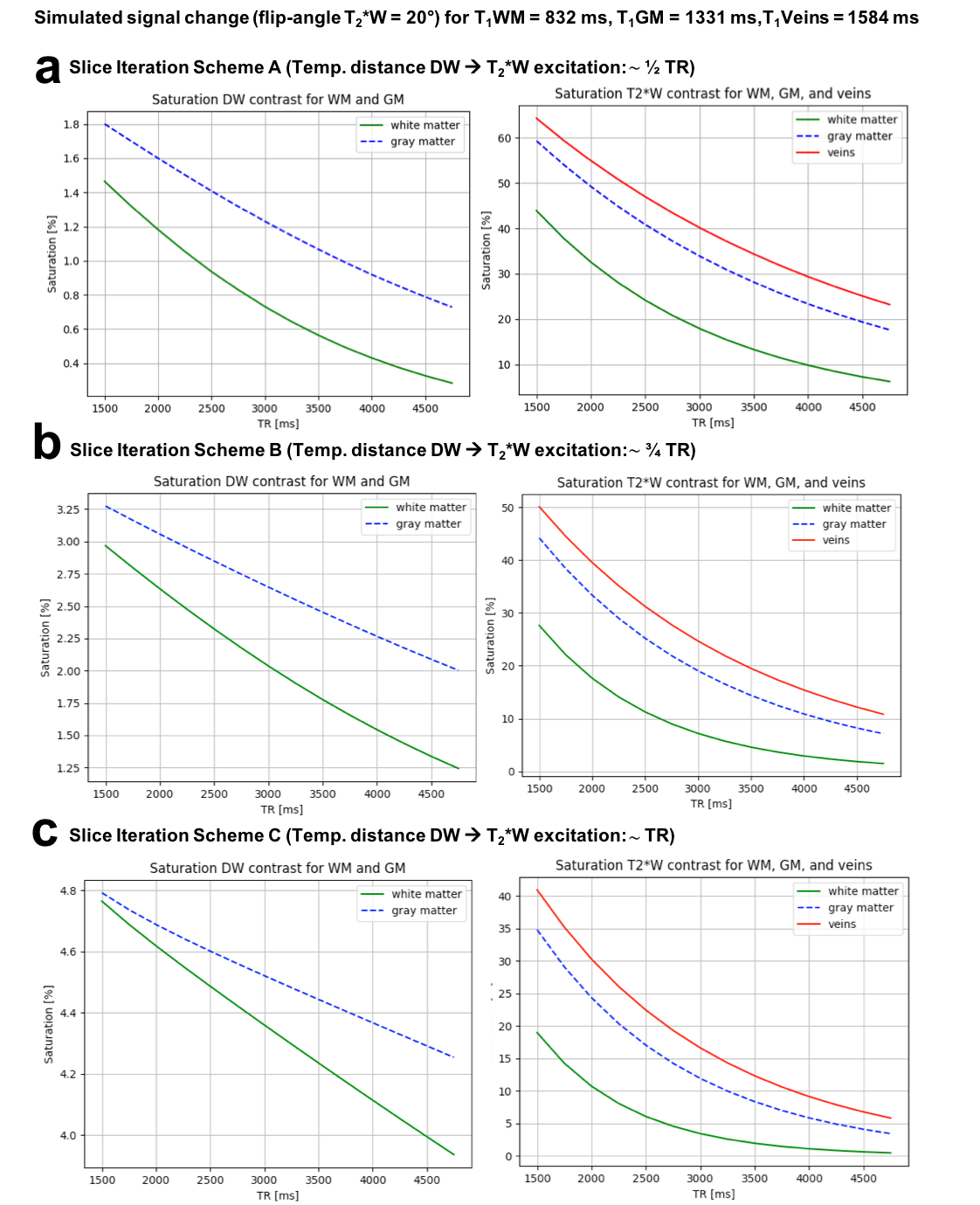


Figure S7:

Results from measurements with TR = 2250 ms


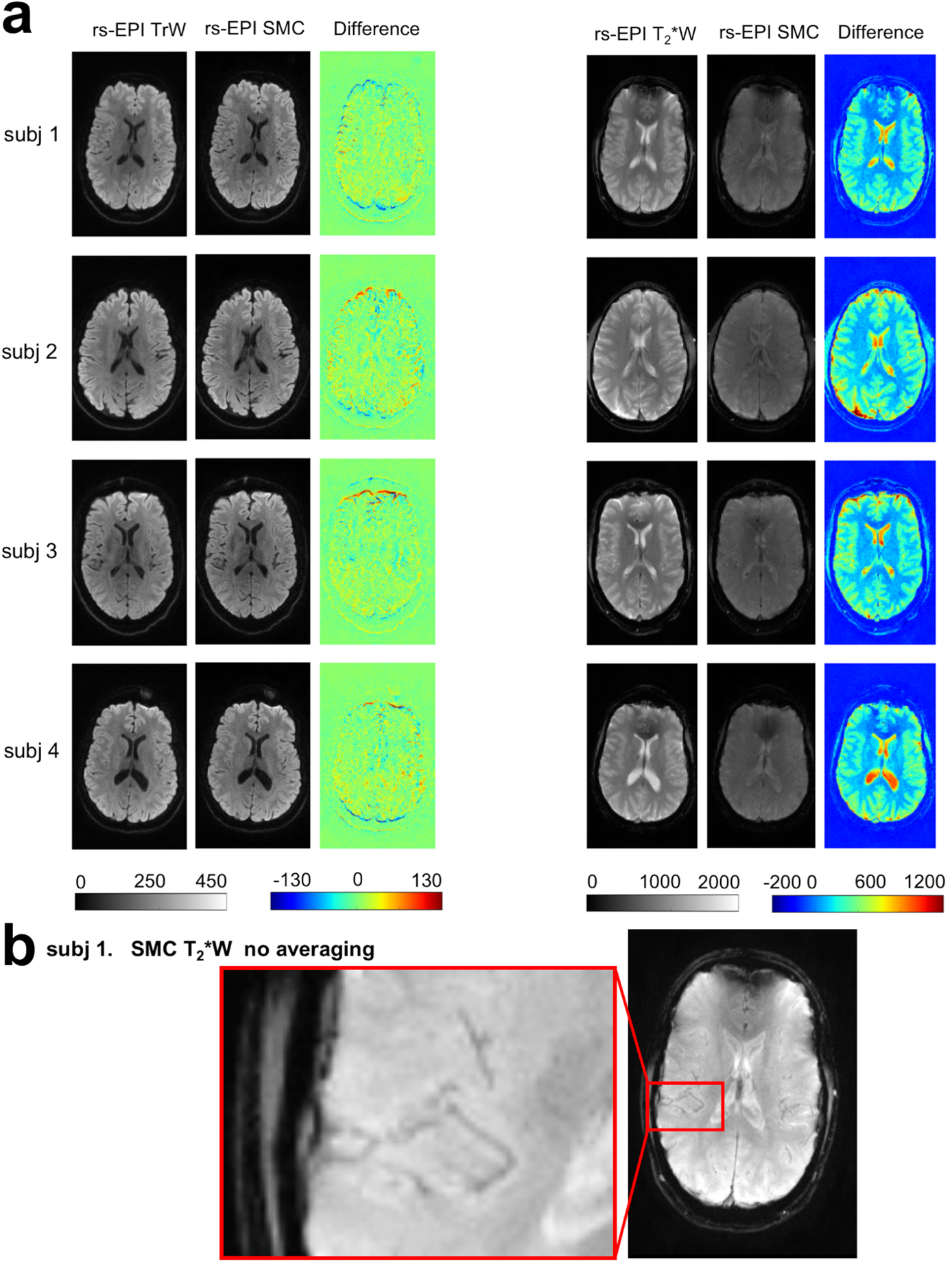


Figure S8:


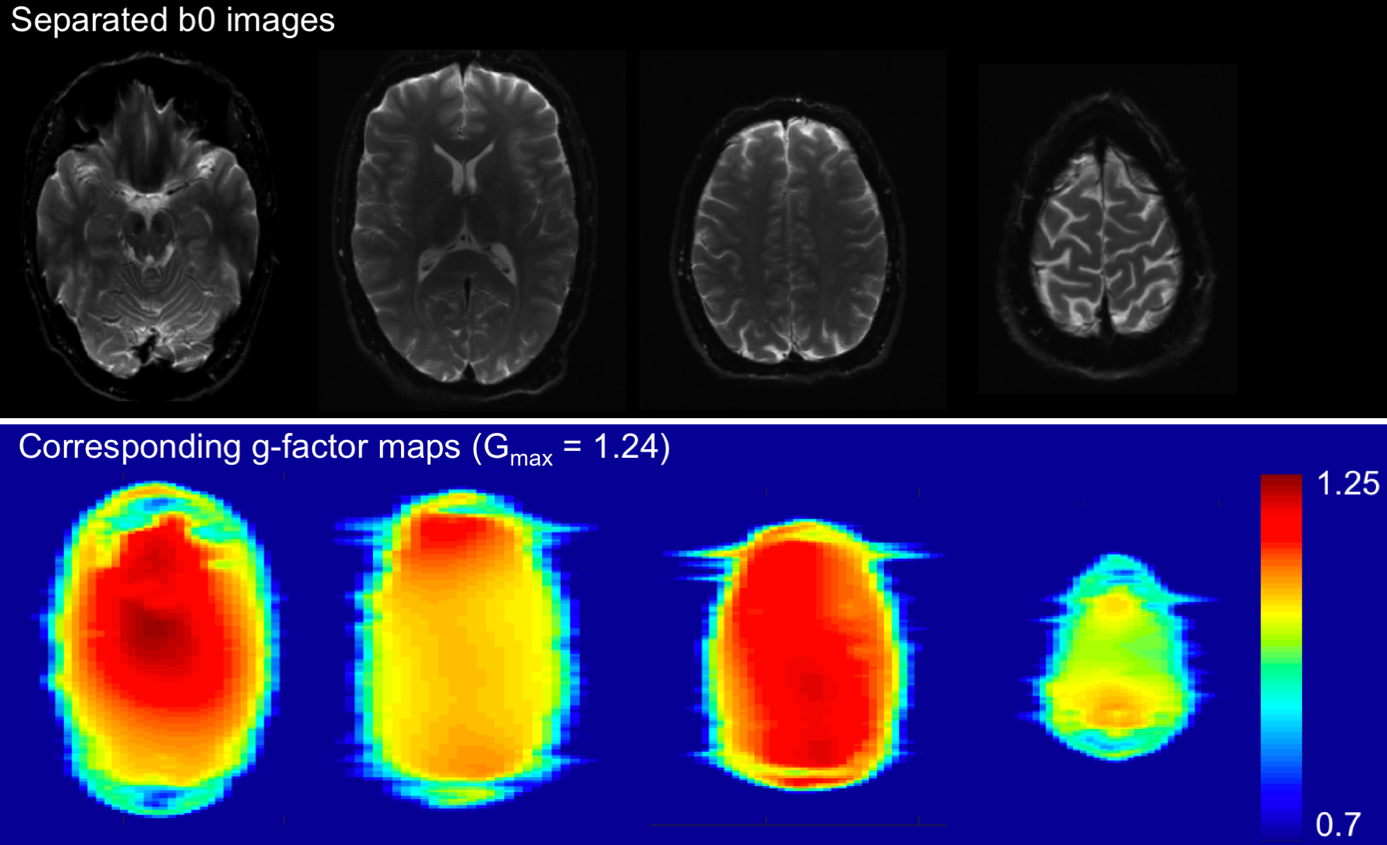

Supplement: Supplementary file 1 — Supplementary file1 (DOCX 25575 KB) [file 10334_2021_976_MOESM1_ESM.docx]
